# Affinity-Bead Assisted Mass Spectrometry (Affi-BAMS): A Multiplexed Microarray Platform for Targeted Proteomics

**DOI:** 10.3390/ijms21062016

**Published:** 2020-03-16

**Authors:** Ghaith M. Hamza, Vladislav B. Bergo, Sergey Mamaev, Don M. Wojchowski, Paul Toran, Camilla R. Worsfold, M. Paola Castaldi, Jeffrey C. Silva

**Affiliations:** 1 Discovery Sciences, BioPharmaceutical R&D, AstraZeneca, Boston, MA 02451, USA; ghaith.hamza@astrazeneca.com (G.M.H.); paola.castaldi@astrazeneca.com (M.P.C.); 2 Molecular, Cellular and Biomedical Sciences, University of New Hampshire, Durham, NH 03824, USA; don.wojchowski@unh.edu (D.M.W.); paul.toran@unh.edu (P.T.); 3 Adeptrix Corporation, Beverly, MA 01915, USA; vbergo@adeptrix.com (V.B.B.); smamaev@adeptrix.com (S.M.); cworsfold@adeptrix.com (C.R.W.)

**Keywords:** targeted proteomics, PTMs, BAMS, bead assisted mass spectrometry, MALDI MS, multiplex assays

## Abstract

The ability to quantitatively probe diverse panels of proteins and their post-translational modifications (PTMs) across multiple samples would aid a broad spectrum of biological, biochemical and pharmacological studies. We report a novel, microarray analytical technology that combines immuno-affinity capture with Matrix Assisted Laser Desorption Ionization Mass Spectrometry (MALDI MS), which is capable of supporting highly multiplexed, targeted proteomic assays. Termed “Affinity-Bead Assisted Mass Spectrometry” (Affi-BAMS), this LC-free technology enables development of highly specific and customizable assay panels for simultaneous profiling of multiple proteins and PTMs. While affinity beads have been used previously in combination with MS, the Affi-BAMS workflow uses enrichment on a single bead that contains one type of antibody, generally capturing a single analyte (protein or PTM) while having enough binding capacity to enable quantification within approximately 3 orders of magnitude. The multiplexing capability is achieved by combining Affi-BAMS beads with different protein specificities. To enable screening of bead-captured analytes by MS, we further developed a novel method of performing spatially localized elution of targets from individual beads arrayed on a microscope slide. The resulting arrays of micro spots contain highly concentrated analytes localized within 0.5 mm diameter spots that can be directly measured using MALDI MS. While both intact proteins and protein fragments can be monitored by Affi-BAMS, we initially focused on applying this technology for bottom-up proteomics to enable screening of hundreds of samples per day by combining the robust magnetic bead-based workflow with the high throughput nature of MALDI MS acquisition. To demonstrate the variety of applications and robustness of Affi-BAMS, several studies are presented that focus on the response of 4EBP1, RPS6, ERK1/ERK2, mTOR, Histone H3 and C-MET to stimuli including rapamycin, H_2_O_2_, EPO, SU11274, Staurosporine and Vorinostat.

## 1. Introduction

Across the diverse disciplines of cell signaling, development, disease and therapeutics, global insight into cellular pathways and molecular mechanisms can now be effectively gained via recent advances in RNAseq and discovery proteomics. For each technology, this includes improvements in efficiency, and deepened coverage, that continue to evolve [1,2,3,4]. For sub-sets of functional effectors uncovered by these discovery approaches, the need frequently arises to focus attention on prioritized sets of target proteins (e.g., specific biomarkers, signal transducers, oncogenes, toxicology markers). This focus often extends to post-translational modifications (PTMs) that are critical for modulating specific sites of activation or inhibition of response pathways. For such protein panels, Western blotting provides a conventional approach to assessing the protein levels and PTMs. In addition, quantitative measurements are improving through features such as in-gel protein standardization [5,6], dynamic imaging [7], and medium-throughput platforms [8]. Throughput, however, remains relatively restricted, as does the ability to multiplex (e.g., via the use of multiple fluorophore detector antibodies [9,10]). For certain defined sets of native proteins, multiplexed immuno-affinity bead technologies also have been developed [11,12], and have expanded from 10- to 20-plex targets, to 100-plex for select predetermined bead sets [13]. This technology, however, has focused predominantly on cytokines and secreted proteins [14,15]. This approach is not easily customizable since it typically involves the use of paired antibodies that first capture, and then detect each immuno-adsorbed target protein of interest (a requirement that can limit the success rate of customized assays). Sensitivity is comparable to ELISA’s, but background and non-specific signals can be generated due to off-target binding that is challenging to assess.

Mass Spectrometry (MS) is one of the few analytical platforms that can quantitatively and specifically measure multiple proteins, together with their constituent peptides (as well as other biomolecules). The proteome’s unmatched complexity, however, imposes a significant bottleneck for MS approaches [16], and the existence of a diverse array of dynamic PTMs presents further challenging attributes to address within multiple model systems. As a multidimensional analytical method, liquid chromatography (LC)-MS/MS is often employed to partition protein complexity within biological specimens, and to enable peptide identification via MS/MS fragmentation patterns [17]. Depending on the complexity of biological samples, chromatographic separation can require between 0.5 and 6 h per sample to separate peptide analytes, and decrease signal interference from wide ranges of constituent proteins [17]. LC-MS/MS can additionally require relatively large amounts of starting sample material to identify and accurately quantify low abundant protein variants (e.g., specific sites of phosphorylation, acetylation, methylation, point mutations). Furthermore, sample preparation workflows can require multi-dimensional separation strategies and/or targeted depletions [18,19,20,21], while subsequent bioinformatic analysis can require deconstruction of chimeric spectra [22] to effectively quantify specific proteins of interest.

To address sensitivity (e.g., for low abundant proteins), and/or high throughput analysis (e.g., for large cohort clinical studies), targeted MS acquisitions such as multiple reaction monitoring (MRM) [23], and parallel reaction monitoring (PRM) [24,25], have been developed. Technical limitations, however, persist including the need for optimized instrument methods for specific precursors [26], limits of on-column injection load and space charge effects [21], ion suppression [27], and (in the case of reporter-based multiplexed experiments) ratio compression can compromise accuracy of the quantitative method [28]. Configuring and implementing a robust MS-based, targeted assay for protein panels of interest therefore remains a major challenge. For targeted proteomic applications, recent studies by Borchers and colleagues have successfully demonstrated the use of immuno-MALDI (iMALDI) by combining immuno-affinity enrichment of peptides followed by MALDI MS analysis as a sensitive, targeted proteomic screening method for clinical biomarkers [29,30,31,32,33]. While enabling, the iMALDI approach is limited in accommodating multiplexed assays. We have now worked to develop an approach to configure multiplexed, targeted proteomic assays of essentially any target protein for which a single antibody is available that binds specifically to a derived proteolytic peptide that is unique to each target protein of interest (called Affi-BAMS). Using MALDI MS for direct measurement readout, any off-target binding of the reagent antibody is effectively avoided (and/or discounted) by monitoring the expected mass of the resulting proteolytic peptide(s).

We further demonstrate that Affi-BAMS is highly effective at quantitating specific PTMs, such as phosphorylation and acetylation, and can also be configured to readily distinguish and quantitatively measure protein isoforms and orthologues. This platform additionally can be readily adjusted from an assay format employing multiple, replicate immuno-affinity beads per sample, to a multiplex assay using a defined panel of immuno-affinity beads (i.e., focused target set) for large numbers of samples. The latter becomes advantageous for monitoring biomarkers, and for high throughput screening applications. This framework serves to introduce our present studies that first report on the workflow for Affi-BAMS, and to highlight method optimization, reproducibility, sensitivity and the application of several standard MS-based approaches to perform quantitative measurements. To demonstrate the diverse and reliable utility of Affi-BAMS, a series of pilot experiments are presented including: the multiple, site-specific phosphorylation of 4EBP1 and RPS6 in response to several stimulatory treatments; kinase inhibitor effects on C-MET; the profiling of select cytokine regulated signal transduction factors; and histone H3 protein modifications upon HDAC inhibition. Taken together, this work underscores the broad-based versatility of Affi-BAMS in (pre-)clinical research.

## 2. Results

### 2.1. Overall Workflow

**Figure 1:**
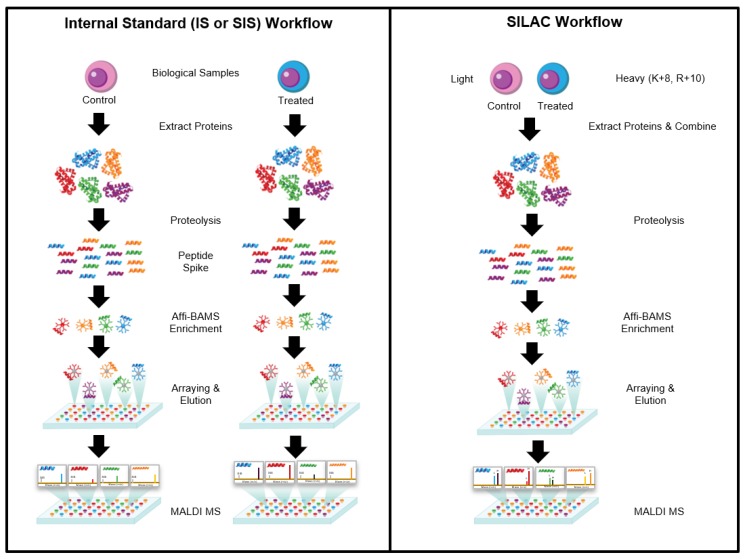
Affi-BAMS Workflow including quantitation via internal standard or SILAC approaches. Affi-BAMS quantification has been validated for robustness and accuracy using traditional mass spectrometry methods: 1) unlabeled, internal-standard (IS) peptides, 2) stable-isotope-labeled internal-standard (SIS) peptides spiked (left panel) and 3) Stable Isotope Labeling with Amino acids in Cell culture (SILAC, right panel). In these workflows, soluble protein is extracted from the biological material and digested with protease into their corresponding peptide fragments. In the internal standard workflow, IS or SIS peptide for the intended target is spiked into the protein digest at a known concentration. Affi-BAMS beads are used to enrich for the corresponding target peptides. Beads are washed and then assembled into an ordered array onto a MALDI slide. The affinity-captured peptide targets are eluted from the beads within each micro-well and deposited onto the microarray slide for MALDI MS acquisition. The MALDI MS spectrum is matched to a reference spectrum for identification of the target protein or protein modification. The MS intensity of the target is then used to determine relative quantitation based on the reference peptide (IS or SIS) or the heavy and light SILAC pairs.

The Affi-BAMS assay workflow combines three common bio-analytical technologies: magnetic beads, microarrays and MALDI TOF MS to uniquely provide scalable detection and quantification of multiple protein targets in a miniaturized, multiplexed single-bead format [34,35,36,37,38]. With a focus on bottom up proteomic applications, the workflow for this platform is outlined in Figure 1. Biological input material is lysed into proteins and digested with a protease; the resulting peptides are captured by antibody-conjugated magnetic beads in a bead suspension format. An array is formed from the reacted beads on a microwell array plate that consists of a flat-surface microscope slide attached to a multi-well silicone elastomer gasket. The bead array is exposed to an aerosol containing solution of a MALDI matrix, which causes the bound peptides to be eluted from the spatially separated beads into their respective microwells. The solvent is allowed to evaporate, which causes the eluted peptides to co-crystallize with the MALDI matrix in spots at the bottom of the microwells, i.e., on the surface of the slide. The silicone gasket is removed to expose the array of spots, and is subsequently measured by MALDI MS and analyzed to identify peptides present in individual spots and to extract quantitative data, which is used to calculate abundance changes of corresponding precursor proteins.

### 2.2. Affi-BAMS Assay Beads and Slide Arrays

**Figure 2:**
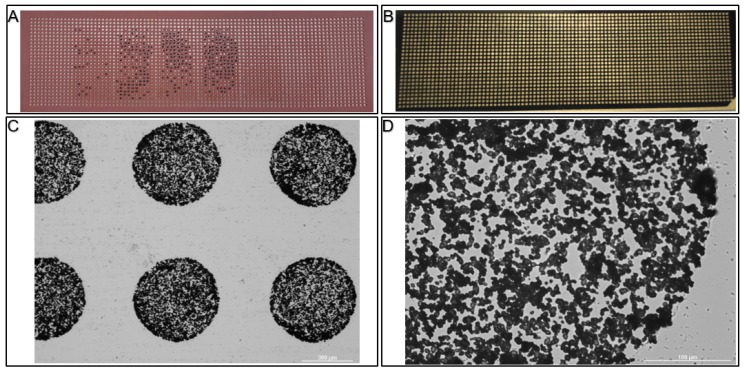
Affi-BAMS Array. An elastomer (Panel **A**) is used to spatially confine each Affi-BAMS bead within one single well. A MALDI matrix sprayer uniformly delivers aerosolized elution buffer to each of the microwells, throughout the entire slide. Once the elution protocol is complete, an array of co-crystalized targets in MALDI matrix is formed, which is subsequently analyzed by MALDI MS (Panel **B**). A bright field image of a section of the Affi-BAMS microarray is shown with CHCA matrix spots produced using the aerosol elution method for matrix deposition. The scale bar in the lower right of the image measures 300 microns (Panel **C**). A magnified image of a single microarray spot is shown to illustrate the uniform coverage and size of CHCA matrix crystals within the spot. The scale bar in the lower right of the image measures 100 microns (Panel **D**).

Each Affi-BAMS bead includes a covalently coupled antibody that is validated to specifically capture a defined proteolytic fragment of a target protein of interest (or PTM). Antibodies used in Affi-BAMS assays are directed against a linear epitope that is either located within an unmodified region of the target protein, or a region of the protein that is subject to a specific post-translational modification. Additional consideration for selecting an Affi-BAMS antibody includes preservation of the epitope when the target protein is hydrolyzed by a selected protease (e.g., trypsin or alternative). The Affi-BAMS’ beads are comprised of agarose and contain a magnetic particle core to assist with bead manipulation during the sample preparation. The beads are produced with a narrow size distribution of 375 µm ± 15 µm. The companion gasket contains microwells with dimensions that are slightly larger than a single bead (i.e., 500 µm depth and diameter) to ensure one bead per well occupancy. The gaskets are produced from acid and organic solvent resistant elastomer, which allows exposure to elution buffers without polymer contamination. As the beads and the gaskets are custom-produced, it is possible to vary the size and packing density of the spots on an Affi-BAMS slide, from about 1000 spots per slide for 900 µm diameter spots (750 µm beads) to over 2000 spots/slide for 500 µm spots (375 µm beads) and over 10,000 spots/slide for 250 µm spots (200 µm beads) (See Figure 2, and Figure S1A). The quality of individual matrix-containing spots is verified by using an optically transparent indium tin oxide (ITO) coated microscope slide for MALDI MS (Bruker Daltonics, Billerica, MA). A matrix solution containing CHCA (5 mg/mL) was applied to a hydrated microwell array using the elution method described in the Methods section. After the slide was dried and the matrix fully crystalized, the gasket was removed and the array of spots was imaged using CYTATION^TM^ 3 multi-mode reader (BioTEck, Winooski, VT) to illustrate the uniform size of matrix crystals in the 500 micron spots (Figure 2C,D). The uniformity of the matrix crystals provides stable MALDI MS measurement of analyte peptide throughout the area of the spot.

### 2.3. Peptide Preparation and Enrichment on Target-Specific Immuno-Affinity Beads, and Bead Multiplexing

For Affi-BAMS assays, peptides are prepared using the following standard bottom-up proteomics methods [39]. Under denaturing conditions, 100–200 µg of total protein extract from a cell culture, tissue, liquid biopsy or any other type of biological material is generated and subjected to reduction, alkylation and proteolytic digestion. For a specific panel of target proteins and/or PTMs, a specified protease(s), (e.g., trypsin, chymotrypsin, ArgC, LysC, AspN, GluC) is then defined that preserves epitopes recognized by each targeting antibody. In some cases, different proteases may be used for the same Affi-BAMS assay if the epitope sequence is preserved during proteolysis. The option to substitute proteases for a given Affi-BAMS assay ensures that epitope sequences are preserved upon proteolysis, and can also allow assays to extend into regions beyond the targeted epitope. Immediately after digestion, proteolytic peptides can be assayed directly for liquid biopsy samples [40,41] or they can be C18 purified for cell line or tissue samples prior to Affi-BAMS enrichment. Purified peptides are lyophilized and stored (−80 °C) until needed for Affi-BAMS assay.

A single-plex Affi-BAMS assay contains one or several (e.g., 2-3 replicate beads) identical beads conjugated to an antibody that recognizes a specific protein target for the purposes of analytical replicates. A multiplexed Affi-BAMS assay contains a mixture of beads conjugated to different antibodies to carry out simultaneous enrichment of multiple protein targets. Either microcentrifuge tubes, or a 96-well plate format can be employed. For a given 200 µm bead, the binding capacity is approximately 1 pmol, and varies with the bead diameter. Affinity capture of target peptides is typically done for 12 h at 4 °C but can be performed within as little as 3 h. Non-specifically bound peptides are removed by a series of brief washes with PBS, ammonium bicarbonate buffer (10 mM, pH 8.0) and deionized water (2 min, 4 °C). To reduce high levels of non-specific, background binding, a high-salt wash (100 mM Tris, pH 8.0 and 1M KCl) is included prior to the series of standard washes listed above.

### 2.4. Bead Arraying, and Conversion to an Array of Targeted Peptide Micro-Spots

The microwell array plate for assaying the reacted beads includes a silicone elastomer gasket containing an array of through-holes that is attached to a conductive (ITO or gold-coated) glass microscope slide. The number of wells exceeds the number of beads used in the affinity assays to ensure that every bead is singularly deposited. The slide assembly (Figure S1B) then adds a matched silicone gasket with 500 µm diameter micro-wells for arraying 375 µm diameter beads. A multi-chamber frame is then placed over the surface and serves to sub-divide sets of microwells for the parallel processing of multiple sample sets (Figure S1C). The number of sections that can be accommodated on a single slide varies between 1 to 64.

To facilitate bead placement into arrays, microwell gaskets are hydrated with deionized water. Washed Affi-BAMS beads containing bound target peptides are transferred into the sample chamber wells using a magnetic bead picker, are dispersed within chamber wells by gentle agitation, and then settled into individual gasket microwells by gravity and brief centrifugation. Next, the sample chamber frame is detached, and bulk water is removed from the array surface using a slide spinner (see Methods).

The bead array is then exposed to an aerosol containing cyano-4-hydroxycinnamic acid (CHCA) in 50% acetonitrile and 0.4% trifluoroacetic acid (TFA), generated by a MALDI matrix sprayer. The aerosol is applied to the surface of the plate with the micro-well gasket and Affi-BAMS beads facing the MALDI matrix sprayer’s airbrush. The matrix sprayer delivers aerosolized elution buffer sequentially row by row to cover the entire microarray. The aerosol method of matrix deposition provides reproducible delivery of small, precisely measured volumes of elution buffer that are uniformly distributed across the entire surface of the microwell plate, without overfilling individual wells [42]. Additionally, the rate of elution buffer application is optimized such that the rate of the solution deposition onto the slide is closely matched to the rate of the solvent evaporation from the slide. The matrix sprayer continues to deliver elution buffer to the surface of the slide for approximately 15 min to ensure efficient release of the captured peptide targets. The low pH of the solution dissociates unique targeted peptides from each Affi-BAMS bead, while the sidewalls of the micro-well gasket prevents the possible diffusion of eluted peptides into adjacent wells. For each Affi-BAMS bead, the sample chamber also creates a partitioned elution vessel, spatially separating each affinity captured-released analyte. Following the elution cycle, any residual solvent evaporates, and the released peptides co-crystallize and are incorporated into MALDI matrix in confined spots at the bottom of the micro-wells (corresponding to the slide surface). Once the matrix is dry, the silicone gasket is lifted off the slide and any remaining dry agarose beads are removed using a gentle burst of compressed air. Via this process, an array of micro-spots containing purified and concentrated target peptides is generated for subsequent MALDI MS measurement (Figure S1A,S2).

### 2.5. MS Data Acquisition, and Data Analysis

Arrays of peptide micro-spots generated via the Affi-BAMS steps (above) next can be measured on essentially any MALDI MS equipped to accept standard 25 × 75 mm microscope slides. The pattern of spots created using the 500 µm diameter micro-well gasket creates an array of 26 × 88 spots (with 0.8 mm spacing between each spot) that can be interrogated via MALDI MS. MS spectra are acquired sequentially from each spot in positive (or negative) ion mode, using linear optics (between 750–7000 m/z) at 15–25% laser power with a resolution of approximately 1000 at 1500 m/z. The laser beam is typically focused to approximately 10–20% of the diameter of the spot to ensure that the individual targets are sampled without contamination from adjacent spots. A MALDI MS instrument operating at 2 kHz speed (2000 spectra/sec) can analyze a single 500 µm spot in less than 1 s and an entire 2288-spot array within approximately one hour.

The mass spectra acquired from individual spots are analyzed for the presence of specific masses that correspond to the expected molecular weights of the proteolytic peptides that were previously validated for each Affi-BAMS assay included in either the single-plex assay or the multi-plex assay. Since the bead array is random, the identity of each eluted peptide target in a particular location is not known until after the MS acquisition. Accordingly, it is obtained by matching the MALDI MS spectrum against the reference database of the pre-validated Affi-BAMS assays included in the experiment. For those Affi-BAMS assays directed toward a specific protein modification, the corresponding PTM localization can be assigned as well, based on the designated specificity of antibody used for the specific Affi-BAMS assay.

Once a peptide analyte within a spot is identified, the corresponding peak intensity (or peak area) is used to quantify the analyte between various conditions. Quantification from the MS signal can be performed using a variety of well-established methods. These methods can include metabolic labeling (such as SILAC) [43,44,45], stable isotope labeled internal standards (SIS) [46,47,48] or addition of non-isotope labeled reference peptide(s). In each case, relative quantitation between multiple conditions is determined by using the internal or spiked standard to normalize the peak intensity (or peak area) of the target analyte. The mass of the captured peptide provides the unique identifier for each target peptide/protein and the relative abundance is determined by comparing the endogenous signal to a reference signal [32,33,49].

### 2.6. Affi-BAMS Specificity and Target Validation

Critical to designing and implementing Affi-BAMS assays is proper validation of the affinity captured peptide target(s). Each Affi-BAMS assay can be validated through MALDI MS in either linear and/or reflector mode, by comparing the observed mass to the predicted peptide mass of the captured proteolytic peptide while considering the antibody specificity to a specific region (or a modification site) within the target protein of interest. In addition, target validation can be confirmed through MS/MS sequencing of the affinity-captured peptide for sequence verification, if needed. For a typical Affi-BAMS assay, we first identify the appropriate proteolytic conditions that would generate corresponding peptide(s), which preserve the epitope region of the intended protein target, as specified by the reagent antibody used for the targeted peptide enrichment. We compare the observed MALDI MS mass measurement(s) to the corresponding theoretical proteolytic peptide masses for the protein of interest. The linear mode MALDI TOF MS acquisition provides the best sensitivity and acquires observed masses that are typically within 0.25 Da of the predicted mass of the intended target peptide with a well calibrated instrument. Although reflector mode MALDI TOF MS is less sensitive, it typically generates mass measurements within 25 ppm of the intended target peptide and provides added specificity when performing identification of the target analyte. An Affi-BAMS assay is validated when there is agreement between the specified digestion conditions, the site or sequence specificity of the antibody, the observed and theoretical mass of the intended target peptide(s), and/or the sequence identification by MALDI MS/MS. After the Affi-BAMS assay has been validated, the linear mode acquisition is sufficient for all subsequent assays and typically provides higher sensitivity for the detection of the target analyte.

**Figure 3:**
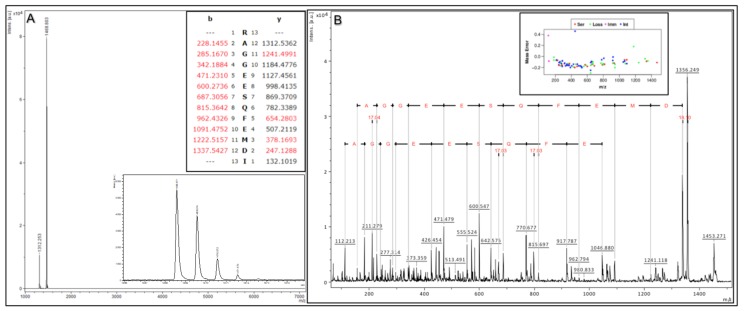
Example validation of 4EBP1 Affi-BAMS Assay. An Affi-BAMS assay for 4EBP1 (total, C-terminus) was performed with untreated MKN45 cell lysate as described in the Methods section. An in silico tryptic digest of 4EBP1 produces the following peptides containing zero and one missed cleavage: 1) R.AGGEE**S**QFEMDI.- and 2) K.RAGGEE**S**QFEMDI.- with S112 highlighted in bold and underlined to emphasize the surrounding region used from the immunizing peptide. The calculated masses (MH^+^, monoisotopic) for each of the peptides listed above are 1312.536 and 1468.637 m/z (z = 1), respectively. A full scan MALDI MS spectrum (750–7000 m/z) was collected in linear mode from the Affi-BAMS assay for the C-terminus of 4EBP1, showing 1468.80 and 1312.25 (m/z, z = 1) as the two most intense peaks (Panel **A**). A full scan MALDI MS spectrum (750–7000 m/z) was collected in reflector mode from the same spot of the Affi-BAMS assay for C-terminus 4EBP1, showing 1468.57 (m/z, z = 1) as the most intense peak. An MS/MS spectrum was collected for the most intense peak, 1468.57 (m/z, z = 1), and was searched through Protein Prospector and identified as the K.RAGGEE**S**QFEMDI.- peptide to 4EBP1. In addition, the MALDI MS data has been annotated to highlight the consecutive b-ions (top series) and internal fragment ions (bottom series) for the identified peptide sequence (Panels **B**). Several examples of ammonia loss are shown with the delta mass of 17 m/z, and addition of water with a delta mass of 18 m/z. The fragment ion at 1453.271 corresponds to the precursor with loss of ammonia. The mass error distribution of the matching fragment ions from the Protein Prospector database search is shown.

To demonstrate the above validation protocol (as performed for each Affi-BAMS assay), we describe a targeted Affi-BAMS assay to the C-terminus of 4EBP1 under tryptic conditions as an example. Control sample was prepared using C18 purified peptides from a tryptic digest of MKN45 cells. An Affi-BAMS assay for 4EBP1 (total, C-terminus) was performed as described in the Methods section. Based on the product description of the antibody used for the Affi-BAMS assay, the monoclonal antibody was directed to unmodified 4EBP1 at the C-terminus of the protein (using a peptide surrounding residues S112 for the epitope (Cell Signaling Technology, 9644S)). An in silico tryptic digest of 4EBP1 produces the zero and one missed cleavage: 1) R.AGGEESQFEMDI.- and 2) K.RAGGEESQFEMDI.- with S112 highlighted in bold and underlined. The calculated masses (MH^+^, monoisotopic) for each of the peptides listed above are 1312.536 and 1468.637, respectively. A full scan MALDI MS spectrum (750–7000 m/z) was collected in linear mode from the Affi-BAMS assay for C-terminus 4EBP1, showing 1312.25 and 1468.80 (m/z, z = 1) as the two most intense peaks (Figure 3A), constituting at least 95% of the total signal intensity in the MALDI MS spectrum. A full scan MALDI MS spectrum (750–7000 m/z) was collected in reflector mode from the same spot of the Affi-BAMS assay for C-terminus 4EBP1, showing 1468.57 (m/z, z = 1) as the most intense peak. An MS/MS spectrum was collected for the most intense peak, 1468.57 (m/z, z = 1), and was searched through Protein Prospector and identified as the K.RAGGEESQFEMDI.- peptide to 4EBP1. In addition, the MALDI MS/MS data has been annotated to highlight the consecutive b-ion series for the identified peptide sequence (Figure 3B) along with the mass error distribution of the matching fragment ions from the Protein Prospector database search. Given the strong agreement between the calculated masses and the observed mass measurements from the MALDI MS and MS/MS to the intended peptide(s) of 4EBP1 by the antibody used for the Affi-BAMS assay, the intended target peptide and protein is validated for subsequent targeted assays to 4EBP1 (total, C-terminus). Future Affi-BAMS assays are acquired in linear mode for ideal sensitivity where expected masses of the target peptide(s) are quantified through their corresponding peak intensity using either an internal or external standard.

### 2.7. Detection and Quantitative Measurements of Protein PTMs

**Figure 4:**
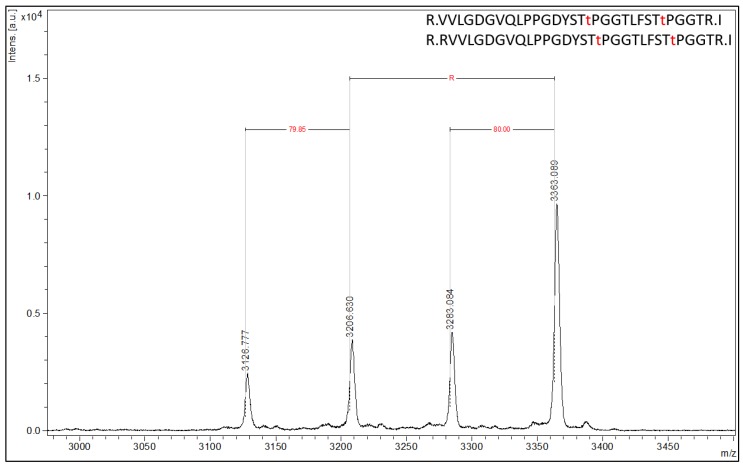
Detection of Singly and Doubly Phosphorylated 4EBP1. Control sample was prepared using C18 purified peptides from a tryptic digest (200 µg) of MKN45 cells (untreated). An Affi-BAMS assay for 4EBP1 (pT37 and pT46) was performed as described in the Methods section. An in silico tryptic digest of 4EBP1 produces the following peptides with either a single phosphorylation (at T37 or T46) or dually phosphorylated peptide (at both T37 and T46) with calculated masses (MH^+^, monoisotopic) for each of the peptides as 3127.498, 3207.465, 3283.599 and 3363.566 m/z (z = 1, MH^+^), respectively. The data collected from the MALDI MS spectrum shows the following measured peptide masses (interpolated monoisotopic) as 3126.78, 3206.63, 3283.08 and 3363.09 m/z (z = 1, MH^+^), respectively. These masses represent the 4EBP1 peptides corresponding to the zero and one missed cleavage product, with the one missed cleavage product corresponding to an additional arginine residue at the N-terminus of the resulting tryptic peptide (delta mass of 156.18 m/z). An 80 m/z mass shift is observed for both cleavage products, corresponding to the singly and doubly phosphorylated forms of each tryptic peptide since the antibody displays cross-reactivity with singly phosphorylated pT37 or pT46.

By employing PTM antibodies, Affi-BAMS further allows quantitation of protein modifications in protein-derived peptides. In this section, three informative examples applications are presented. The first demonstrates a validated Affi-BAMS assay and antibody for pT37 and pT46 phospho-sites of 4EBP1. Based on the product description, this monoclonal antibody is directed to dually phosphorylated 4EBP1 at T37 and T46 (and detects endogenous levels of 4EBP1 only when phosphorylated at T37 and/or T46 (Cell Signaling Technology, 2855S)). Using C18 purified peptides from a tryptic digest of MKN45 cells, we performed an Affi-BAMS assay for 4EBP1 (pT37 and pT46) and acquired a MALDI MS spectrum for the captured and eluted target peptides (Figure 4).

An in silico tryptic digest of 4EBP1 produces the following peptides with either a single phosphorylation at T37 or T46 (highlighted in bold only) or dually phosphorylated at both T37 and T46 (highlighted in bold and underlined): 1) R.VVLGDGVQLPPGDYST**T**PGGTLFST**T**PGGTR.I, 2) R.VVLGDGVQLPPGDYST**T**PGGTLFST**T**PGGTR.I, 3) R.RVVLGDGVQLPPGDYST**T**PGGTLFST**T**PGGTR.I and 4) R.RVVLGDGVQLPPGDYST**T**PGGTLFST**T**PGGTR.I. The calculated masses (MH^+^, monoisotopic) for each of the peptides listed above are 3127.498, 3207.465, 3283.599 and 3363.566 m/z (z = 1, MH^+^), respectively. The Affi-BAMS data collected from the MALDI MS reports on the following measured peptide masses (interpolated monoisotopic): 3126.78, 3206.63, 3283.08 and 3363.09 m/z (z = 1, MH^+^). These masses represent the 4EBP1 peptides corresponding to the zero and one missed cleavage products (listed above), with the one missed cleavage product corresponding to an additional arginine residue at the N-terminus of the resulting tryptic peptide (delta mass of 156.18 m/z). Notably, an 80 m/z mass shift is observed for both cleavage products, corresponding to the singly and doubly phosphorylated forms of each trypsin cleavage product. This accurately reflects this antibody’s cross-reactivity with 4EBP1 forms that are singly phosphorylated at T37 and T46 that share a STTPGGT motif.

As a second illustration of using Affi-BAMS to monitor PTMs, we used a validated Affi-BAMS assay for phospho C-MET dually phosphorylated at both pY1234 and pY1235. In MKN45 cells, signaling pathways for growth and survival are known to depend on C-MET (MET-driven), and exhibit elevated phosphorylation of C-MET levels at Y1234 and Y1235 [50]. Using C18 purified tryptic peptides from MKN45 cells, we performed an Affi-BAMS assay for C-MET pY1234 and pY1235, and acquired a MALDI MS spectrum for the affinity captured, and eluted target peptide(s) (Figure S3). An in silico tryptic digest of C-MET produces the following peptides from zero to two missed cleavages: 1) K.E**YY**SVHNK.T, 2) K.E**YY**SVHNKTGAK.L and 3) R.DMYDKE**YY**SVHNK.T with pY1234 and pY1235 highlighted in bold and underlined. The calculated masses (MH^+^, average) for each of the peptides listed above are 1200.087, 1557.499 and 1852.815 m/z, respectively. The data collected from the MALDI MS spectrum shows a single, dominant peak at 1853.21 m/z corresponding to the two missed cleavage products listed above that is presumably due to the presence of KE and also the dually phosphorylated pY1234 and pY1235 residues that would normally force missed cleavages [51].

In a third example application, several proteins can be highly modified in their PTM’s, and this is often the case for ribosomal proteins, and histones. In an Affi-BAMS assay, we predict these species can be resolved by using an antibody that is capable of capturing a designated region of such proteins and monitoring the presence of the various PTMs by their corresponding unique mass measurements. We demonstrated this application in an Affi-BAMS assay specific to RPS6 pS235 and pS236 in order to monitor the effects of oxidative stress on the phosphorylation status of RPS6′s C-terminal tail [52]. Here, MKN45 cells were treated for 20 min with +/− 2 mM H_2_O_2_ (see Methods). We then digested protein lysates from control and treated MKN45 cells with trypsin and carried out an Affi-BAMS assay for RPS6 pS235 and pS236 to generate MALDI MS spectra for [+] and [−] H_2_O_2_ exposure conditions (Figure S4). Comparing the MALDI MS spectra from each condition, a series of common peaks were observed between 2000 and 2500 m/z, with some distinct differences among the relative peak intensities between control DMSO vs 20 min peroxide treatment. Focusing first on the MALDI MS spectrum from the DMSO (Figure S4A), we observe the following series of common masses annotated from the interpolated mono-isotopic mass measurements using SNAP2 peak detection algorithm available in FlexAnalysis: 2011.08, 2090.90, 2168.03, 2170.69, 2247.16, 2327.07 and 2407.27 m/z. These masses correspond to a series of multiply phosphorylated derivative peptides surrounding the target region of the antibody (pS235 and pS236). The first series of masses are associated with the base sequence that spans amino acids 223–249, R.RL**SS**LRASTSKSESSQK.-, containing two (2011.915 MH^+^), three (2091.776 MH^+^) and four (2171.798 MH^+^) phosphorylated residues (with the pS235 and pS236 highlighted in bold and underlined as the target site of the antibody). The second series of masses are associated with the base sequence that spans amino acids 222–249, R.RRL**SS**LRASTSKSESSQK.-, containing two (2168.337 MH^+^), three (2248.100 MH^+^), four (2328.034 MH^+^) and five (2407.774 MH^+^) phosphorylated residues (with the pS235 and pS236 highlighted in bold and underlined). The MALDI MS spectrum from the DMSO treatment shows the RL**SS**LRASTSKSESSQK peptide containing between two and four phosphorylated residues (with two of the phosphorylation sites required at S235 and S236, as determined by the specificity of the antibody). In addition, the DMSO treatment also shows the RRL**SS**LRASTSKSESSQK peptide (at a lower intensity) containing between two and four phosphorylated residues. Looking at the 20 min peroxide treatment (Figure S4B), there is an increase of the phosphorylation status of the C-terminal peptide. The MALDI MS spectrum for the RL**SS**LRASTSKSESSQK peptide shows predominantly between 3 to 5 phosphorylated residues, with the relative intensity of the dually phosphorylated species substantially lower than what is observed in the DMSO. Similarly, the MALDI MS spectrum for the RRL**SS**LRASTSKSESSQK peptide also shows higher order phosphorylation (between three and five phosphorylated residues) as observed from the relative intensities of the corresponding masses and the reduction of the dually phosphorylated form of the peptide. Although the MALDI MS spectrum initially appears complicated, in the context of the Affi-BAMS assay directed to RPS6 (pS235 and pS236), the annotation of the peaks is straightforward when using the details related to the specificity of the RPS6 (pS235 and pS236) antibody. The modulation of phosphorylation upon stress conditions (peroxide treatment) is quite clear and can be easily monitored using the Affi-BAMS assay.

**Figure 5:**
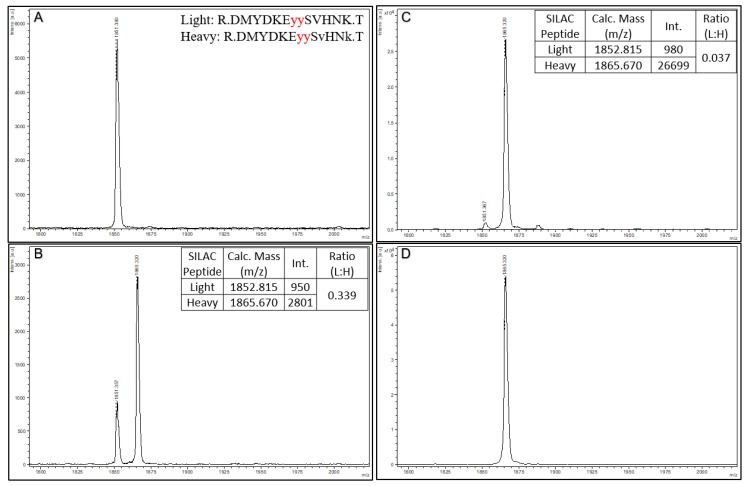
Affi-BAMS Assay for C-MET (pY1234 and pY1235) with SIS Spike. (**A**) 0 fmoles, (**B**) 100 fmoles, and (**C**) 1000 fmoles of SIS peptide in 100 µg of MKN45 tryptic peptide digest. An additional sample was prepared containing only 1000 fmoles of SIS peptide alone, with no MKN45 tryptic peptides (**D**). The expected mono-isotopic masses for the endogenous and SIS peptides are noted as 1852.815 m/z and 1865.670 m/z, respectively. Endogenous (light) and SIS (heavy) C-MET phosphopeptides for the 1000 (L:H ratio = 0.037) and 100 (L:H ratio = 0.339) fmole spike conditions show agreement with the expected fold-change comparison between the two spiked peptide level conditions (calculated fold-change = 9.24, expected fold-change = 10.0).

To further demonstrate the quantitative aspect of Affi-BAMS, we use a SIS spiked reference peptide for the dually phosphorylated C-MET phosphopeptide (pY1234 and pY1235) with the following sequence: DMYDKE**YY**SvHNk. In this SIS phosphopeptide standard, the phosphotyrosines are highlighted in bold and underlined, and heavy amino acids are lower case (valine = v + 6 and lysine = k + 8). This yields a theoretical monoisotopic mass of 1865.670 MH+ (average mass of 1866.815 MH^+^). Three aliquots of trypsin digested MKN45 cell lysate were prepared (100 µg) for a C-MET pY1234 and pY1235 Affi-BAMS assay, and aliquots were spiked with either 0, 100 or 1000 fmoles of the SIS peptide (see Methods). An additional sample was prepared containing only 1000 fmoles of SIS peptide alone, with no MKN45 tryptic peptides (see Methods). After performing the Affi-BAMS assay for the C-MET phosphopeptide samples, the MALDI MS spectra shown in Figure 5 were obtained. As predicted, we observe the endogenous C-MET phosphopeptide in the three samples containing the tryptic peptides of MKN45 (theoretical monoisotopic mass of 1851.670 MH^+^ and average mass of 1852.815 MH^+^ with a sequence of DMYDKE**YY**SVHNK). We also observed the SIS peptide at 1865.670 MH^+^, which was spiked at levels of 1000 and 100 fmol. Comparing the intensity ratios between the light and heavy C-MET phosphopeptides for the 1000 (L:H ratio = 0.037) and 100 (L:H ratio = 0.339) fmol spike conditions, we observe a corresponding fold-change that is in agreement with the two spiked peptide levels (calculated fold-change = 9.24, theoretical fold-change = 10.0). In the sample that did not contain spiked SIS standard, no signal contribution for the theoretical mass of the SIS peptide was observed. Similarly, we do not observe any signal contribution for the mass of the endogenous peptide in the SIS only sample.

### 2.8. Affi-BAMS Sensitivity and Reproducibility

**Figure 6:**
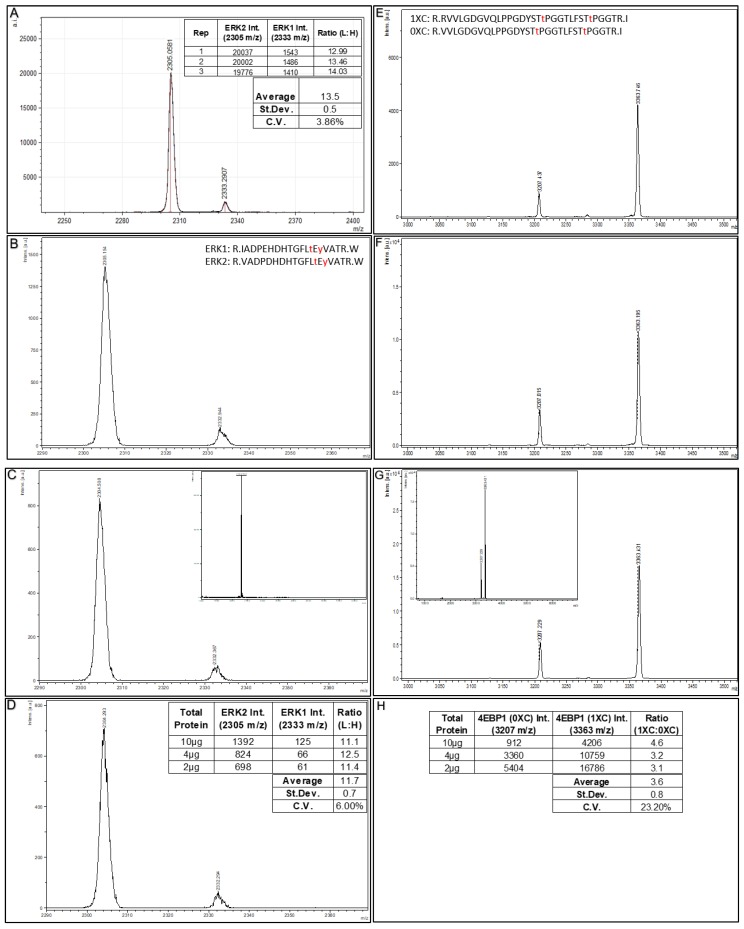
Affi-BAMS Assay Reproducibility and Sensitivity. Three replicate Affi-BAMS assays were conducted for ERK1 (pT202 and pY204) and ERK2 (pT185 and pY185) phosphopeptides using a total of 50 µg of trypsin digested soluble protein from HeLa cells treated with 2 mM H_2_O_2_ for 20 min (**A**). The Affi-BAMS assay captures both ERK1 and ERK2 peptides on the same bead to measure both peptides within a single MALDI MS spectrum. The expected monoisotopic masses for the ERK2 and ERK1 peptides are 2303.937 and 2331.968 m/z (z = 1), respectively. In an Affi-BAMS assay with three replicate beads, an average ratio of 13.5 was observed between ERK2 versus ERK1, with a CV of 3.86% among the three calculated ratios. In a separate experiment, an Affi-BAMS assay was performed for ERK1 and ERK2 using a total of 10.0, 4.0 and 2.0 µg of trypsin digested protein (**B**–**D**). The calculated intensity ratios (ERK2:ERK1) show an average ratio of 11.7 with a CV of 6.0%. (**C**) shows an inset image of the full scan MALDI MS with little to no background signal from the ERK1/2 Affi-BAMS assay. An Affi-BAMS assay was performed for 4EBP1 (pT37 and pT46) using a total of 10.0, 4.0 and 2.0 µg of trypsin digested protein (**E**–**G**). The expected monoisotopic masses for the zero and one missed cleavage tryptic peptides for 4EBP1 (pT37 and pT46) are 3207.465 and 3363.566 m/z (z = 1), respectively. The ratio between the zero and one missed cleavage products (1XC:0XC) from each of the three different sample amounts were calculated to be an average of 3.6 with a CV of 23.2% (Panel **H**). (**G**) shows an inset image of the full scan MALDI MS with little to no background signal from the 4EBP1 Affi-BAMS assay.

With respect to the amount of starting material needed for Affi-BAMS assays, we demonstrate the ability to work with as little as 2µg of total starting protein. Here, we performed several Affi-BAMS assays using decreasing amounts of total soluble protein from digested cell culture lysates ranging from 50µg to 2µg. First, we show the MALDI MS signals generated from three replicate Affi-BAMS assays for ERK1 and ERK2 within the same tryptic digest using 50 µg of total digested protein per bead assay (Figure 6A). The cell lysate used in this experiment is from HeLa cells treated with H_2_O_2_ (2mM, 20min). The Affi-BAMS assay was performed using a monoclonal antibody that recognizes the conserved (pT)E(pY) motif located in both ERK(1 and 2) isoforms and captures (on the same Affi-BAMS bead) the corresponding tryptic peptides within ERK1 (pT202 and pY204) and ERK2 (pT185 and pY187). Under tryptic digestion conditions, the ERK2 tryptic phosphopeptide (R.VADPDHDHTGFL**T**E**Y**VATR.W, pT185 and pY187) has a theoretical monoisotopic mass of 2303.937 m/z and the ERK1 tryptic phosphopeptide (R.IADPEHDHTGFL**T**E**Y**VATR.W, pT202 and pY204) has a theoretical monoisotopic mass of 2331.968 m/z (phospho-threonine and phospho-tyrosine are highlighted in bold and underlined). Based on the validation experiments for this Affi-BAMS assay, we expect both ERK1 and ERK2 tryptic peptides to be captured on the Affi-BAMS bead. Based on Affi-BAMS assays performed with equimolar ratios of synthetic ERK1 and ERK2 peptides (data not shown), we expect the relative abundance of the MALDI MS signal to reflect the relative endogenous levels of ERK1 (pT202 and pY204), and of ERK2 (pT185 and pY187). Moreover, we predict ERK1 (pT202 and pY204) phosphopeptide to be present at much lower levels than the ERK2 (pT185 and pY187) phosphopeptide, consistent with what was observed in vivo for the relative endogenous levels of ERK1 and ERK2 in cell line models [53]. For the observed intensities of the ERK1 and ERK2 MALDI MS signals generated, consistent intensity ratios are generated among the three replicate Affi-BAMS assays (with an average ratio of 13.5, and a coefficient of variance of 3.86% for these analytical replicates).

To further illustrate the sensitivity of Affi-BAMS, we performed assays using the above HeLa cell sample (tryptic digest, peroxide treated) using as little as 2µg of total soluble protein. In Figure 6 (B–D), we show Affi-BAMS assays for both ERK1 (pT202 and pY204) and ERK2 (pT185 and pY187) using 10µg, 4µg and 2µg of this sample. The calculated intensity ratios (ERK2:ERK1) from the MALDI MS signal for ERK1 (pT202 and pY204) and ERK2 (pT185 and pY187) show an average ratio of 11.7 (coefficient of variation of 6.0%). In a related experiment to illustrate method reproducibility, Figure 6E–G shows the Affi-BAMS assays for 4EBP1 (pT37 and pT46) using matched decreasing amounts of total soluble digested protein from peroxide treated HeLa cells (10 µg, 4 µg and 2 µg). From the validation experiments for the 4EBP1 (pT37 and pT46) Affi-BAMS assay, we expect to monitor the following two tryptic peptides from this Affi-BAMS bead: R.VVLGDGVQLPPGDYST**T**PGGTLFST**T**PGGTR.I and R.RVVLGDGVQLPPGDYST**T**PGGTLFST**T**PGGTR.I with the corresponding calculated masses, 3207.464 and 3363.565 (MH^+^). These peptides correspond to the zero and one missed cleavage (0XC and 1XC) products from the tryptic digestion and are reproducibly observed at the same relative ratio. The relative intensity ratios (1XC:0XC) from the MALDI MS signal for the two peptides from the 4EBP1 (pT37 and pT46) Affi-BAMS assay show an average ratio of 3.6 with a CV of 23.2% (Figure 6H). Taken together, Affi-BAMS assays allow for low sample input amounts, without sacrifice in the precision and quantification.

The detection and quantification of low abundant proteins can be challenging using liquid chromatography workflows that require specified dimensions and properties for the analytical column used to separate peptide analytes. One major constraint, that can often complicate monitoring of low abundant analytes, is the binding capacity of the analytical column. For LC-MS workflows, nano-LC chromatography is often used to obtain maximum sensitivity for peptide analytes, using analytical columns that are approximately 50–150 microns in diameter, with a typical recommended binding capacity of only 1.0–3.0 micrograms of total soluble digested protein (from a tissue, biofluid, or cell digest). Liquid biopsy samples, including serum and plasma, have a wide and dynamic range of endogenous proteins, with a small number of proteins representing a large fraction of the total protein mass [54]. For example, the top twenty proteins in serum/plasma represent 95% of the total protein mass. Removal of these proteins from the sample using depletion columns can provide up to a 50-fold increase in protein load to enhance the detection of lower abundant proteins in serum/plasma and decrease the dynamic range [55,56]. Methodologies have also been implemented for deep proteomic analysis such as multi-dimensional protein identification technology (MudPIT) [57,58]. Unfortunately, the depletion methods can lead to loss of other proteins, including low abundant proteins that may be of interest, due to the non-specific binding of protein-protein interactions to proteins that are immobilized on the affinity capture column, and deep proteomic analysis using multidimensional fractionation methods can span several days of instrument time.

As an immunoaffinity-based platform, Affi-BAMS does not rely on chromatographic separation and is not limited by the quantity of input material. Therefore, the quantification of low abundant proteins that are difficult to detect within biological fluids can be achieved by increasing the amount of input material in order to expose a sufficient amount of target peptide for affinity capture on the Affi-BAMS bead. To demonstrate this application, we proteolytically digested 100 µL of human serum (~7000 µg of total soluble protein) with trypsin and performed an Affi-BAMS assay for 4EBP1 (see Methods), to probe the presence of this low abundant protein in serum. From the MALDI analysis, we obtained both MS and MS/MS spectra to validate the presence of 4EBP1 in the human serum sample (Figure S5). While it is known through RNA methods that 4EBP1 is present in a wide range of blood related cells (https://www.proteinatlas.org/ENSG00000187840-EIF4EBP1/blood), it has not previously been detected by either immunoassays, mass spectrometry or proximity extension assays (to the best of our knowledge). The ability to increase low abundant protein signal for quantification through Affi-BAMS, without the need to deplete highly abundant proteins, provides new opportunities for monitoring biomarkers in liquid biopsy samples for disease biology, drug development research, and clinical diagnostic screening.

### 2.9. Expanding Peptide Coverage Using Complementary Proteases

In bottom-up proteomics, trypsin is the most widely used protease for converting proteins into peptides for subsequent LC-MS analysis [59]. Trypsin is a robust protease that cleaves at the carboxyl side of arginine and lysine residues, and tolerates many denaturing conditions required to help expose soluble proteins for reduction, alkylation and make accessible for proteolytic cleavage. With an average molecular weight of tryptic peptides from an in-silico digest of the human proteome being approximately 1800 Da, the predicted average tryptic peptide length is 18 amino acids [59,60]. By using alternate proteases, the average mass of the resulting proteolytic peptides can be shifted to a greater value, resulting in longer peptide sequences. With this flexibility, proteolytic conditions can be customized to retain and expose the specific region of interest for essentially any target protein. This provides several options for generating a specific sequence of amino acids required for Affi-BAMS assays. To demonstrate this application, an MKN45 cell lysate was digested independently with either trypsin or chymotrypsin, and the resulting proteolytic peptides were independently processed using an Affi-BAMS assay for 4EBP1 (total, C-terminus). The Affi-BAMS assay for 4EBP1 (total, C-terminus) has been validated to capture the following peptides under tryptic digestion conditions: 1) R.AGGEE**S**QFEMDI.-, 2) K.RAGGEE**S**QFEMDI.- and 3) R.NSPEDKRAGGEE**S**QFEMDI.-, with calculated masses (MH^+^, monoisotopic) for each of the peptides being 1312.536, 1468.637 and 2138.929, respectively. A full scan MALDI MS spectrum (750 – 7000 m/z) was collected in linear mode from the Affi-BAMS assay for 4EBP1 (tryptic conditions), showing 1312.25 and 1468.80 (m/z, z=1) as the two most dominant peaks (Figure S6A) as expected for the tryptic peptide for the zero and one missed cleavage products. Under chymotryptic conditions, we observe the following two peptides from the 4EBP1 (total, C-terminus) Affi-BAMS assay: L.RNSPEDKRAGGEE**S**QFEMDI.- and L.RN**S**PEDKRAGGEE**S**QFEMDI.-, with the calculated masses (MH^+^, monoisotopic) of 2295.030 and 2374.997, respectively. A full scan MALDI MS spectrum (750 – 7000 m/z) was collected in linear mode from the Affi-BAMS assay for 4EBP1 (chymotryptic conditions), showing 2294.41 and 2374.41 (m/z, z = 1) as the two most predominant peaks (Figure S6B), corresponding to the chymotryptic peptide with and without phosphorylation at Serine-101. Localization of phosphorylation at Serine-101 is evident since it is the only amino acid that is able to be phosphorylated within the extended sequence, RN**S**PED, compared to the resulting sequence from the tryptic digest which did not show any evidence of phosphorylation (Serine-112). Since MALDI MS spectrum from the tryptic digest did not contain any additional mass measurements corresponding to phosphorylation of the R.AGGEESQFEMDI.- to K.RAGGEE**(pS)**QFEMDI.- (with the addition of +80 Da), the phosphorylation must reside upstream on Serine-101. Interestingly, phosphorylation of 4EBP1 at S101 is a known phosphorylation site on 4EBP1 that is required for the release of 4EBP1 from eIF4E for subsequent initiation factor complex assembly [61]. By using different proteases for the preparation of varying length peptides for a given Affi-BAMS assay, we demonstrate how one can explore adjacent regions of a target protein for permutations such as PTMs (i.e., phosphorylation, acetylation, methylation) or even single amino acid point mutations (illustrated later).

We demonstrate the analytical rigor and specificity of Affi-BAMS through the use of several validated assays for 4EBP1, C-MET, ERK1 and ERK2. To demonstrate the diverse utility of Affi-BAMS for both basic and (pre-)clinical research, several example pilot experiments are presented to identify and quantify the (1) inhibition of C-MET through the use of kinase inhibitors, (2) effects of rapamycin on mTOR signaling pathway, and (3) signal transduction in Jak/Stat pathway upon erythropoietin challenge. Furthermore, we demonstrate the ability to monitor point mutations, frequently observed in drug resistance, and introduce a novel method to examine chromatin biology and cross talk of PTMs, frequently found on histones, via a combinatorial histone code approach.

### 2.10. Quantitation via SILAC: Kinase Inhibitors

**Figure 7:**
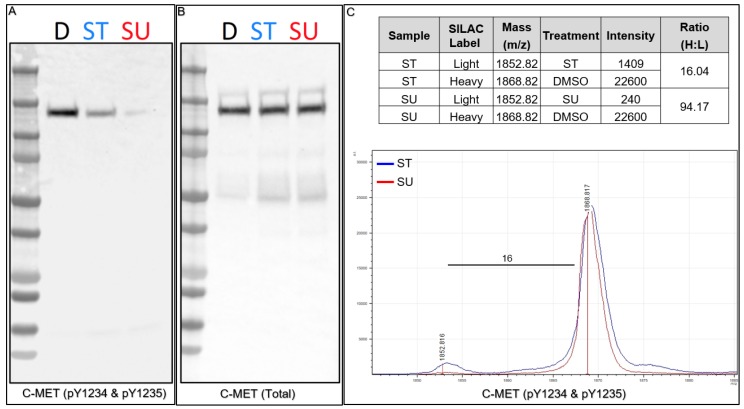
C-MET Inhibition upon ST and SU treatment in MKN45 Cells. MKN45 cells were treated with 0.2 μM Staurosporine (ST, protein kinase C [PKC] inhibitor) or 1.0 μM SU11274 (SU, C-MET inhibitor) for 2 h. The Western blot for dually phosphorylated C-MET (pY1234/pY1235) shows reduction of phosphorylation with ST treatment and a more dramatic decrease with SU treatment, compared to the control (D, DMSO) condition (**A**). In addition, there is no significant change in the amount of total C-MET among the control and treatment conditions (**B**). An Affi-BAMS assay for C-MET (pY1234 and pY1235) was conducted on equally combined MKN45 SILAC labeled pairs for both treatments (SU: red and ST: blue) and the corresponding MALDI MS spectrum from each assay is overlaid (normalizing the intensities to the DMSO channel) to illustrate the relative quantification of C-MET upon ST and SU treatment conditions (Panel **C**). A significant decrease of the dually phosphorylated C-MET is observed upon both treatments. The relative fold-change was calculated from the normalized intensities from the corresponding light (ST or SU) and heavy (DMSO) masses of the affinity captured C-MET peptides. The SU treatment shows greater inhibition (94.17, SU:D) of phosphorylation on C-MET (pY1234 and pY1235) relative than with ST treatment (16.04, ST:D) and is consistent with the results from the Western blot.

As SIS peptides may not always be readily customized to perform quantitative experiments on targets of interest for a highly multiplexed Affi-BAMS assays using a cell line model, we demonstrate that a classical light and heavy **S**table **I**sotope **L**abeling by/with **A**mino Acids in Cell Culture (SILAC) experiment is well suited for quantitative Affi-BAMS analyses [45]. To demonstrate this application, we cultured MKN45 (a C-MET driven cancer cell line) under light and heavy SILAC conditions (light = kinase inhibitor, heavy = DMSO; K+8 and R+10) and treated cells with either 0.2 μM Staurosporine (ST, protein kinase C [PKC] inhibitor) or 1.0 μM SU11274 (SU, C-MET inhibitor) for 2 h (see Methods) as described by Stokes and colleagues [18]. We performed Western blots for both total C-MET and phospho-C-MET (pY1234 and pY1235) to confirm inhibition by both kinase inhibitors prior to performing subsequent Affi-BAMS assays (Figure 7A,B). Consistent with previous reports, upon ST treatment we observed a decrease in phosphorylation with phospho-C-MET (pY1234 and pY1235) and a more significant decrease upon SU treatment. Total C-MET levels, in contrast, were consistent throughout the control and treatment conditions (as observed by Stokes and coworkers) [18]. After confirming consistency between control Western blots and the previous work, we combined protein lysates from the light and heavy SILAC pairs (1:1, by total protein mass) and prepared the material for Affi-BAMS assay to monitor phospho-C-MET (pY1234 and pY1235) levels in response to treatment with ST or SU inhibition. The resulting MALDI MS spectrum shows the expected masses for both the light (1851.669, calculated monoisotopic MH^+^) and the heavy (1867.669, calculated monoisotopic MH^+^) form of the dually phosphorylated tryptic C-MET target peptide (R.DMYDKE**YY**SVHNK.T). The light (L, kinase inhibitor treated, ST or SU treated) and heavy (H, control, DMSO treated) pairs show inhibition of the dually phosphorylated activation loop site of C-MET upon SU and ST treatment. Based on the relative intensity ratio of the light and heavy SILAC pairs, we observe a 94-fold decrease in signal upon SU treatment, and a 16-fold decrease upon ST treatment relative to the control (H:L). The results we obtained from the Affi-BAMS assay are consistent with our control Western blot data and also with the previous studies that were performed using immuno-affinity LC-MS methods, where the authors reported a higher degree of inhibition by SU treatment of phosphorylation at the activation loop of C-MET (pY1234 and pY1235). In addition, the results for the inhibition of C-MET (pY1234 and pY1235) by SU and ST were also consistent with previous Affi-BAMS experiments that were performed using standard methods for normalization of MALDI MS signal with an external spiked peptide [34].

### 2.11. Quantitation via SILAC: mTOR Inhibitor

**Figure 8:**
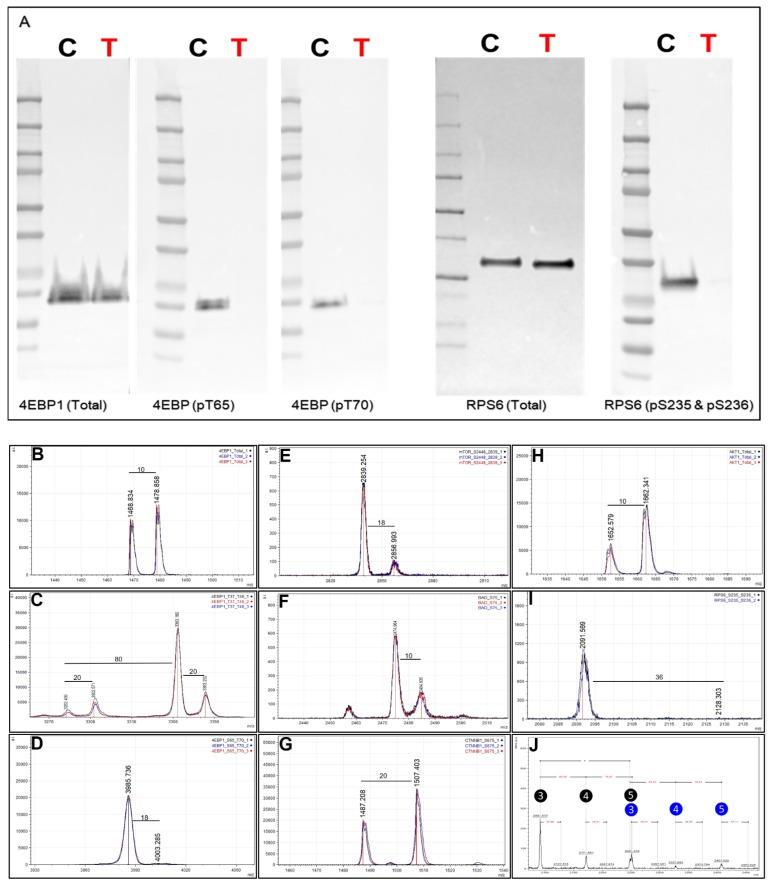
Rapamycin treatment in MKN45 Cells. MKN45 cells were SILAC labeled (light = DMSO, heavy = rapamycin; K + 8 and R + 10) and treated with either DMSO or 1mM rapamycin for 2 h. Western blot for total RPS6 and 4EBP1 show no change in total protein amount; however, a significant inhibition in phosphorylation is observed on both proteins (**A**,**C**) = Control [DMSO]; T = Treated [1mM rapamycin]). A multiplexed Affi-BAMS assay was conducted for total and phospho specific sites for mTOR, 4EBP1, AKT1, RPS6, CTNNB1 and BAD (**B**–**J**). Mass shift due to the heavy labeling of residues is notated as well as phosphorylation shifts. Raw files were imported into mMass and normalized between replicates on the most intense peak to generate ratios between the replicate light and heavy SILAC pairs. (**J**) demonstrates the triple- (3), quadruple- (4) and penta- (5) phosphorylated tryptic peptides of RPS6 without (black) and with (blue) missed cleavage.

To further demonstrate SILAC quantitation with the Affi-BAMS workflow, we examined the response of the human gastric carcinoma cell line, MKN45, to rapamycin (an inhibitor of mTOR signaling). mTOR acts as a sensor of mitogen, energy and nutrient levels and is a regulator of cell growth and autophagy. Dysregulation of mTOR signaling has been implicated in cancer, diabetes, obesity, neurological diseases and genetic disorders [62]. Inhibition of mTOR by rapamycin inhibits mTORC1, and mTORC1 modulates downstream phosphorylation of 4EBP1 and therefore the phosphorylation of a downstream target RPS6, both involved in protein translation. MKN45 cells were cultured in SILAC media (light = DMSO control and heavy = inhibitor treated) and treated with either control (DMSO) or rapamycin (1mM) to monitor endogenous protein changes (total and phosphorylation) using a multiplexed Affi-BAMS assay (see Methods). First, we confirmed the effects of rapamycin treatment through Western blot by screening for total and phosphorylated RPS6 (pS235 and pS236) as well as for total and phosphorylated 4EBP1 (pS65/pT70) (Figure 8A). As expected, we did not observe a dramatic change of total protein levels between control and rapamycin treatment conditions for either RPS6 and 4EBP1; however, a significant decrease in phosphorylation levels was observed for both 4EBP1 (pS65 and pT70) as well as for RPS6 (pS235 and pS236). After confirming expected protein/PTM changes by Western blot, we proceeded to use the SILAC labeled MKN45 cells for a subsequent, multiplexed Affi-BAMS assay containing some common target proteins evaluated in the Western blot analysis as well as for additional protein targets that were expected to be modulated by rapamycin treatment.

As Affi-BAMS provides immuno-affinity enrichment for specific target(s) using one bead per target, we illustrate how the assay platform can be expanded to carry out a multiplexed assay by combining several established Affi-BAMS assays and co-incubating the corresponding beads with purified peptides from a tryptic digest of the equally combined MKN45 SILAC sample. Individual, validated Affi-BAMS assays were combined and used for the purpose of conducting a targeted, multiplexed assay to monitor an established assay panel of proteins/PTMs (see Methods). The peptide/protein targets included in the Affi-BAMS assay were the following: 4EBP1 (total, C-terminal), 4EBP1 (pT37 and/or pT46), 4EBP1 (pS65 and T70), AKT1 (total, C-terminal), BAD (pS75), CTNNB1 (pS675), mTOR (pS2448) and RPS6 (pS235 and pS236). The relative fold-change between control and rapamycin treatment is reported based on the matching SILAC peptide pairs (heavy:light, rapamycin:DMSO) for all targeted assays (Table 1). The quantitative results of the Affi-BAMS assay are generated from the intensity ratios of the SILAC pairs observed from the corresponding MALDI MS signal of each targeted assay. Overall, the quantitative changes we observe from the control Western blots correlated well with the quantitative fold-changes we determined from the MALDI MS signal of the Affi-BAMS assays. The Affi-BAMS results define a dramatic decrease in the phosphorylation of RPS6 (S235 and S236) and 4EBP1 (both T35 and T46 as well as S65 and T70) upon rapamycin treatment (Figure 8C,D,I,J). The Western blots show no significant changes in total protein for both RPS6 and 4EBP1 (Figure 8A), which correlates with the Affi-BAMS assay for total 4EBP1 (C-terminus) showing only a modest increase between control and treatment conditions (1.22-fold, Figure 8B).

While an Affi-BAMS assay for total RPS6 is not yet available, we are able to monitor other protein targets, certain of which are known to be affected by rapamycin treatment. Among these, decreased phosphorylation of mTOR on S2448 is observed (0.15-fold, Figure 8E), in keeping with mTOR as a target of rapamycin inhibition [63]. In addition, we observe a modest increase in AKT1 (total, C-terminus) levels of 2.89-fold (Figure 8H) and an increase in phospho-CTNNB1 at S675 (1.73-fold, Figure 8G). Interestingly, the Affi-BAMS MALDI MS spectrum of 4EBP1 (pT37 and pT46) shows a set of SILAC pairs corresponding to the singly (pT37 or pT46) and doubly phosphorylated (p37 and p46) 4EBP1 tryptic peptides with sequences R.RVVLGDGVQLPPGDYST**T**PGGTLFST**T**PGGTR.I, representing the single missed cleavage tryptic peptide. Here, two arginine residues explain the 20 Da (2R+10) mass difference between these light and heavy peptide pairs (Figure 8C). Based on observed mass measurements and corresponding SILAC ratios from the MALDI MS spectrum, an increase is observed for singly phosphorylated 4EBP1 (pT37 or pT46, 3.06-fold) together with parallel decrease in doubly phosphorylated 4EBP1 (pT37 and pT46, 0.25-fold). The ability to independently yet simultaneously monitor single and doubly phosphorylated sites of 4EBP1 at T37 and T46 is a unique capability of the Affi-BAMS assay platform, and is enabled by the clean capture and resolution of target peptides by MALDI MS. Using Western blot or sandwich ELISA-based methods, the signal for singly and doubly phosphorylated 4EBP1 would be observed as a single band or overlapping signal. Figure 8J further shows the MALDI MS spectrum obtained from the Affi-BAMS assay of RPS6 (pS235 and pS236). The mass spectrum shows the presence of triple-, quadruple- and penta-phosphorylated tryptic peptides of RPS6, each of which exhibits dramatic decrease upon comparing the corresponding light and heavy mass of the SILAC pairs. This level of detail is absent from the corresponding Western blot of the same sample (Figure 8A) using the same RPS6 (pS235 and pS236) antibody, with only a single protein band resolved at the expected molecular weight of RPS6 (28.7 kDa).

### 2.12. Quantitation via SILAC: Erythropoietin Signal Transduction Factors

**Figure 9:**
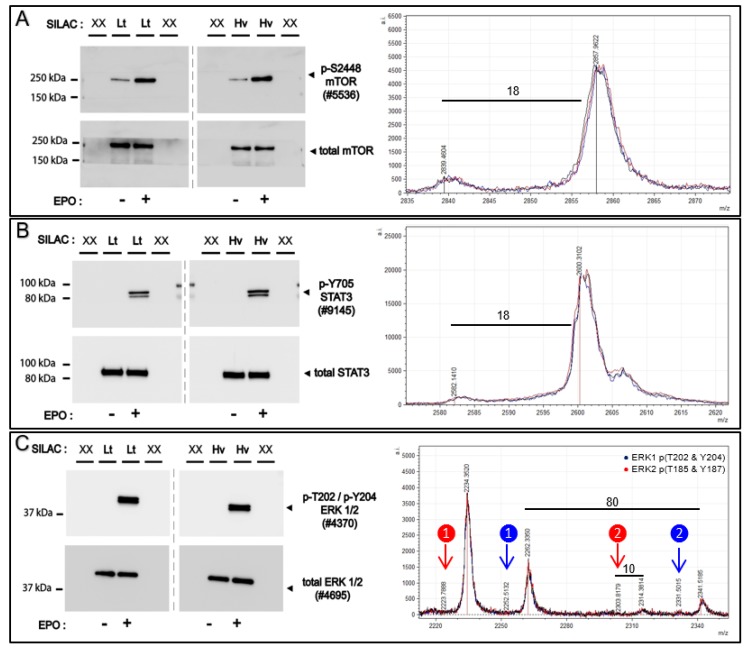
EPO Challenge in UT7epo-E cells. SILAC labeled UT7epo-E cells (forward labeled [light= -EPO, heavy= +EPO] and reverse labeled [light= +EPO, heavy= -EPO]; K + 8 and R + 10) were challenged with +/- 5U/mL EPO for 15 min. Western blots were conducted to validate target’s response for both light and heavy SILAC labeled cells as well as reverse labeled cells to confirm Affi-BAMS assays’ results (Lt = Light, Hv = Heavy, XX designates empty lane). The MALDI MS spectrum for select targets in the multiplexed Affi-BAMS assay are shown for the forward labeled pair, as well as the western blots for mTOR (Panel **A**), STAT3 (Panel **B**), and ERK1/2 (Panel **C**). The mass shift due to the incorporation of one heavy arginine is noted (+10) as well as the appropriate mass shift for phosphorylation (+80) that are found on the ERK1 and ERK2 peptides (see Table 2 for additional details). The red circles indicate the masses for the singly (labeled “1”) and doubly (labeled “2”) phosphorylated peptides for ERK2. The blue circles indicate the masses for the singly (labeled “1”) and doubly (labeled “2”) phosphorylated peptides for ERK1. Raw files were imported into mMass and normalized between replicates on the most intense peak to generate ratios between the light and heavy SILAC pairs.

Affi-BAMS is employed to analyze select signal transduction factors (STFs) that are activated via site-specific phosphorylation in the EPOR/JAK2 system. Erythropoietin (EPO) activates signal transduction through EPOR/JAK2 which regulates multiple downstream signaling pathways including STAT5, PI3K/AKT, NF-kB and RAS-MAPK to modulate the survival, and proliferation and formation of erythroid cells. For Affi-BAMS experiments, we employ EPO-dependent UT7epo cells as a cell model to investigate phosphorylation modulation and signal transduction of select targets including ERK1 (T202 and Y204) / ERK2 (T185 and Y187), STAT3 (Y705), mTOR (S2448) and RPS6 (S235 and S236). The target peptide/protein sites were chosen as known components of EPOR/JAK2 signaling pathways. For select targets, Western blotting additionally was performed. Following transient EPO withdrawal, SILAC labeled UT7epo cells were challenged with +/−EPO (5 U/mL). At 15 min +/−EPO exposure, cells were harvested from both conditions (both forward and reverse SILAC labeling, see Methods). Aliquots of forward and reverse SILAC labeled cells were used for Western blotting to monitor proteins modulated by EPO challenge. The light and heavy SILAC cell lysates from the forward labeling conditions (light = −EPO, heavy = +EPO) were combined evenly (1:1, by total amount of soluble protein) and were subsequently digested with trypsin for Affi-BAMS assays (Figure 9 and Figure S7). From the Western blot results, we observe clear changes in phosphorylation for the proteins that we monitored via Affi-BAMS assays. These include, ERK1 (pT202 and pY204), ERK2 (pT185 and pY187), STAT3 (pY705), mTOR (pS2448) and RPS6 (pS235 and pS236). In contrast, the total levels of these same proteins showed little to no change due to EPO (or SILAC labeling). Although the Western signal for BAD (pS75) was low, there was a clear detectable increase in phosphorylation upon EPO stimulation. Having confirmed the appropriate response from the +/-EPO treatment conditions from the Western blots, we performed the multiplexed Affi-BAMS assay for the selected targets and acquired the corresponding MALDI MS spectrum for each targeted assay (Figure 9 and Figure S7). The relative fold-change between control and EPO treatment was reported based on the matched SILAC peptide pairs (heavy: light, +EPO:−EPO) for all of the targeted assays (Table 2). The heavy labeled amino acid residues (bold blue, K + 8 or R + 10) and phosphorylated S or T amino acid residues (lower case red) are annotated within Table 2 for each of the corresponding Affi-BAMS assays. The quantitative results of the Affi-BAMS assay were generated from the intensity ratios of the corresponding SILAC pairs, which correlated well with all example Western blots. The Affi-BAMS results show dramatic changes in phosphorylation levels for ERK1 (pT202 and pY204), ERK2 (pT185 and pY187), STAT3 (pY705), mTOR (pS2448), RPS6 (pS235 and pS236) and BAD (pS75) upon EPO treatment and are all consistent with the Western blot results. Interestingly, due to the nature of the high resolution of the MALDI MS data, there is a greater amount of information obtained from the ERK1 (pT202 and pY204) and ERK2 (pT185 and pY187) Affi-BAMS assay. The mass measurement from the MALDI MS spectrum provides independent readout for both dually phosphorylated tryptic peptides of ERK1 (pT202 and pY204) and ERK2 (pT185 and pY187). In addition, due to the specificity of the antibody used for the ERK1 (pT202 and pY204) and ERK2 (pT185 and pY187) Affi-BAMS assay, the MALDI MS spectrum also provides readout for the singly phosphorylated tryptic peptides for both ERK1 (pT202) and ERK2 (pT185). Based on the SILAC ratios, we observe a major increase for both singly and dually phosphorylated ERK1 and ERK2 upon EPO stimulation. Equally, we observe the zero and one missed cleavage product peptides of BAD (pS75), both showing similar levels of modulation between control and treatment conditions. It is noteworthy to point out that the SILAC pairs for the two tryptic peptides for BAD (pS75) show the expected mass difference based on the number of constituent lysine’s (K + 8) and arginine’s (R + 10). Unlike the Western blot for BAD (pS75), the signal-to-noise from the MALDI MS data is quite high (>100:1). Moreover, in the Western blot, there is a significant molecular weight change when there is the addition of phosphorylation compared to the typical molecular weight of BAD. Although this correlates with published material where the molecular weight change is seen when phosphorylation is present, this can nonetheless be misconstrued. Because of the expected mass differences between SILAC pairs-based off the predicted target sequence, the SILAC experiments we performed served as a valuable resource to facilitate the validation of the Affi-BAMS assays described in this work. 

### 2.13. Quantitation via Stable Isotope Standard

**Figure 10:**
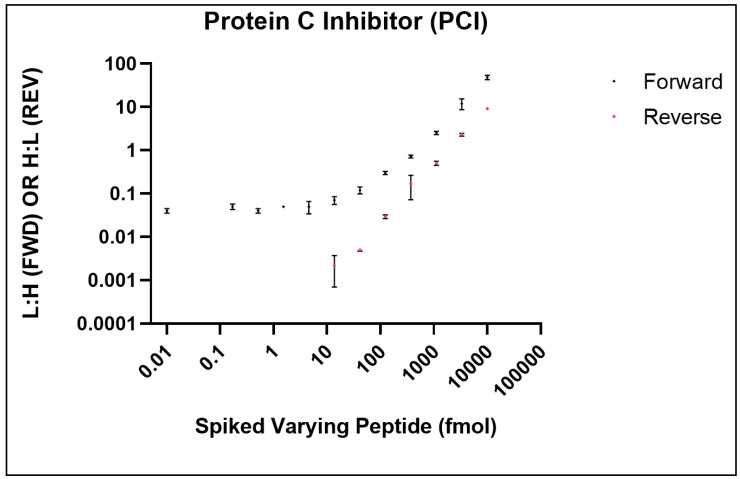
Concentration of PCI using SISCAPA. LOD and LOQ for PCI peptide, EDQYHYLLDR, from human plasma was quantified using Affi-BAMS. The first calibration curve, forward curve, was generated by spiking a constant amount of the heavy peptide (1000 fmoles/well) in a background of digested pooled plasma with a serial dilution of synthetic light peptide to generate a 12-point curve, with the light peptide being titrated from 10,000 fmol to 0.17 fmol (3-fold serial dilution; with no synthetic light peptide in the 12th sample). The reverse curve was generated by spiking constant concentration of the light peptide (1000 fmol) and 3-fold dilutions of the heavy peptide (from 10,000 fmol to 0.06 fmol). The LOD (14 fmoles) was defined as the lowest spiked concentration of SIS peptide that was identifiable in at least two of the three replicates in the experiments. The LOQ (41 fmoles) was defined as the lowest concentration of the analyte that was identifiable in at least two of the three replicates and with a CV of < 30%.

For use in LC-based and chromatography-free platforms, SISCAPA Assay Technologies have developed stable isotope biomarker standards and anti-peptide antibodies for use in subsequent mass spectrometry-based quantification [40,41]. Using SISCAPA’s stable isotope standards for protein C inhibitor (PCI, P05154), we successfully adapted the SISCAPA assay on the Affi-BAMS platform to monitor the PCI peptide, EDQYHYLLDR (Figure 10) [35]. To determine the limit of detection (LOD) and limit of quantification (LOQ) for monitoring PCI by Affi-BAMS, two calibration curves were generated in a digested pooled plasma (10 µL) as described by Razavi et al. [40,41]. The first calibration curve (standard addition curve), was generated by spiking a constant amount of the heavy peptide (1000 fmoles/well) in a background of digested pooled plasma together with a serial dilution of synthetic light peptide. The light peptide was titrated from 10,000 fmol to 0.17 fmol (3-fold serial dilution); with no light peptide in a 12th sample. The forward curve reported on the endogenous concentration of the PCI target analyte in the digested pooled plasma (~15 fmol, concentration at the plateau of the forward curve). A reverse curve was also generated by spiking constant concentration of the light peptide (1000 fmol) and 3-fold dilutions of the heavy peptide (10,000 fmol to 0.06 fmol). This curve was used to determine the LOD and LOQ of the assay with respect to the endogenous concentration of PCI. Results demonstrate over 3 order-of-magnitude dynamic range for the EDQYHYLLDR PCI peptide (Figure 10). The LOD (14 fmoles) was defined as the lowest spiked concentration of SIS peptide that was identifiable in at least two of the three replicates in the experiments. The LOQ (41 fmoles) was defined as the lowest concentration of the analyte that was identifiable in at least two of the three replicates and with a CV of <30%. For the measured PCI peptide, the median variation for all the analytical triplicates was approximately 20% CV. There are two points in the reverse curve that show higher levels of variability. These errors can be explained by either pipetting error, insufficient MS data sampling or quantification at low signal-to-noise at the lowest concentration of spiked peptide. Taken together, these results demonstrate the use of stable isotope methods as a mode of quantitation for Affi-BAMS assays.

### 2.14. Expanded Affi-BAMS Applications: Monitoring Protein Point Mutations

The ability to employ an efficient affinity capture platform, in place of LC separation, provides opportunities to consider additional proteomic applications that have typically been difficult to execute. Affi-BAMS assays could be applied to the proteomic characterization of any target protein, PTM profile or multiplex protein panel. To demonstrate this extended capability, we used a validated Affi-BAMS assay targeting 4EBP1 to monitor the presence of a C-terminal missense mutation. Soluble protein was prepared from HCT116 and MKN45 cells, and tryptic peptides were generated for Affi-BAMS assays. HCT116 is known to contain a heterozygous 4EBP1 missense mutation as A107V, therefore, both wildtype and point mutated 4EBP1 become targets in this experiment [64]. Figure S8 shows the MALDI MS spectrum from the Affi-BAMS assay for 4EBP1 C-terminus from MKN45 cells and HCT116. The HCT116 spectrum contains two expected major peaks: the mass for the wild-type tryptic peptide for 4EBP1, K.RAGGEESQFEMDI.-, measured at approximately 1468.637 MH^+^ (calculated monoisotopic mass), and a second peak that corresponds to the tryptic peptide containing the A107V missense point mutation K.R**V**GGEESQFEMDI.- (highlighted in bold and underlined, i.e., the heterozygous copy of 4EBP1), a calculated monoisotopic mass of 1496.669 MH^+^. Since the epitope for the C-terminus antibody relies on the sequences between amino acids 109 – 118 flanking the site of interest, each sequence is captured indiscriminately. Additionally, the MS intensities of the tryptic peptides for wild-type and A107V 4EBP1 are similar, suggesting that they are present at equal levels (assuming the A107V missense mutation does not markedly alter ionization properties relative to wild-type sequence). This experiment highlights the utility of antibodies that are directed to unmodified protein regions and used to monitor regions upstream or downstream of the epitope sequence.

### 2.15. Expanded Affi-BAMS Applications: Exploring Chromatin Biology

Chromatin biology and epigenetics are emerging areas of life science research due to their fundamental role in understanding cell development, regulation of heritable genomic and proteomic attributes and disease progression. Modifications on histones serve as one prime mechanism to regulate the epigenetic machinery through PTM combinatorial diversity that governs gene expression, DNA repair and chromatin packaging. Advances in mass spectrometry instrumentation as well as development of efficient sample preparation methods for histones and PTMs has made it possible to routinely monitor these dynamic changes. LC-MS methods have been developed for bottom-up analysis of histones, which include isolation of nucleosomes and chemical derivatization of the resulting proteolytic peptides to make them amenable for optimal RP-LC separation and subsequent MS analysis [65,66]. Here, we demonstrate the use of Affi-BAMS as a bottom-up method for targeted proteomics of histones.

**Figure 11:**
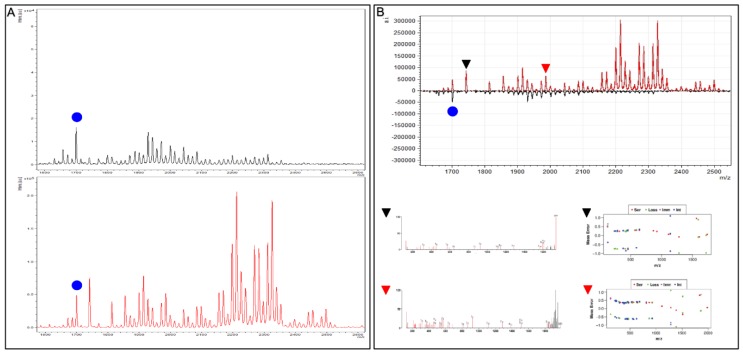
Affi-BAMS assay for H3 (K9-acetylation) in U2OS cells upon SAHA treatment. MALDI MS spectra from an Affi-BAMS assay for H3 (K9-acetylation) in U2OS cells upon DMSO control (**A**, Top) and 5 µM SAHA treatment for 24h (**B**, bottom). The spectra were normalized against a common H3 derived peptide (MH^+^ = 1700.398 [blue circle]) and overlaid in mMass to reflect the relative abundance. Delta change of 14, 42 and 80 m/z are observed accounted by the hyper modified regions within H3 (methylation, acetylation and phosphorylation). There is an increase in overall signal compared to the control as well as a shift in higher molecular weight species suggesting an increase in cross talk of PTMs as well as higher order acetylation. An MS/MS was obtained and searched through Protein Prospector to identify two peaks (1740.97 [black triangle] and 1984.28 [red triangle] m/z). The following peptides from H3 were identified: QTARK(Acetyl)STGGK(Acetyl)APRK(Acetyl)Q and TK(Methyl)QTARK(Acetyl)STGGK(Acetyl)APRK(Acetyl)Q, respectively.

This Affi-BAMS assay is configured using site-specific, PTM antibodies for specific histones of interest, to monitor not only the site of interest but also adjacent sites that are often modified. In this study, we used U2OS cells treated for 24h with either DMSO or Vorinostat (“SAHA”, 5 µM). SAHA is a well-characterized histone deacetylase (HDAC) inhibitor that leads to accumulation of acetylated histone residues [67]. U2OS cell lysates were digested with chymotrypsin and Affi-BAMS assays for acetylated K9 on histone H3 were performed directly from the purified peptide (without nucleosome enrichment or propionlyation). The MALDI MS spectra shown in Figure 11 resolves a pattern of peaks that contain a delta mass corresponding to 14, 42 and 80 m/z values. As expected, the highly modified regions of histone H3 are represented, indicating the presence of diverse peptide variants due to the combination of methylation (+14 m/z), phosphorylation (+80 m/z) and acetylation (+ 42 m/z). Affi-BAMS assay (H3K9 acetylation) captures a unique set of histone H3 marks on a single bead as dictated by the specificity of the site-specific PTM antibody. The enriched peptide variants are resolved within each MALDI MS spectrum, showing a unique pattern of masses that are specific to the proteolytic digestion conditions and the resulting peptide sequence. For some applications, the relative intensities among the pattern of histone H3 masses is a sufficient means to monitor the changes between treatment conditions (as observed for the DMSO vs SAHA treated cells).

In practice, a single reference mass that is common between the two conditions can be used as an internal standard to normalize peak intensities between the control and treated samples. The corresponding peak ratios can then be used to perform a quantitative comparison between different treatment conditions. Using this strategy, we normalize both spectra to the 1700.40 m/z peak. Delta change of 14, 42 and 80 m/z are observed accounted by the hyper modified regions within H3 (methylation, acetylation and phosphorylation). There is an increase in overall signal compared to the control as well as a shift in higher molecular weight species suggesting an increase in cross talk of PTMs as well as higher order acetylation. An MS/MS was obtained and searched through Protein Prospector to identify two peaks (1740.97 [black triangle] and 1984.28 [red triangle] m/z). The following peptides from H3 were identified: K.QTARK(Acetyl)STGGK(Acetyl)APRK(Acetyl)Q.L and R.TK(Methyl)QTARK(Acetyl)STGGK(Acetyl)APRK(Acetyl)Q.L, respectively.

## 3. Discussion

Proteomic analysis of complex biological samples requires the ability to monitor and differentiate a wide range of diverse analytes, which includes (multiple) PTM sites, proteoforms, and point mutations. This frequently presents challenges for analytical technologies that rely on indirect detection methods such as polymerase chain reaction (PCR), proximity extension assay (PEA) or chemiluminescence (ELISA-based assays) [68]. We report a novel analytical immunoassay platform termed Affi-BAMS that combines single affinity bead analyte capture with MALDI MS in a microarray format. This unique platform provides rapid, sensitive, specific, and reproducible quantitative data for protein and PTM targets of interest. This technology therefore successfully addresses the need for accurate profiling of specific targets within complex proteomes including proteoforms, amino acid mutations, and PTMs that otherwise is possible only via time consuming and multi-dimensional LC-MS systems.

Until recently, the power of bead-based assays with respect to multiplexed immuno-affinity analysis has been realized primarily via fluorescence-based flow cytometry methods. By contrast, Affi-BAMS provides detection via MALDI MS thereby eliminating crosstalk of reagents while simultaneously increasing the multiplexing capacity. Thanks to this increased multiplexing, the Affi-BAMS platform can be effectively used in discovery workflows; these attributes provide a unique targeted discovery workflow, which is extended by the ability to readily add antibodies that recognize shared epitopes present in several target proteins. This allows analyses of signaling pathways that can be incorporated into a discovery, multiplexed screen [69]; examples include ERK1/2, JAK/STAT and mTOR signaling via 4EBP1. Importantly, targeted high-throughput screens can be effectively conducted using stable isotope internal standards or non-isotope internal reference standards. Adding either of these approaches transforms the platform into a scalable quantitative method that allows one to compare relative target intensities between samples within large cohorts. This is advantageous when screening for disease biomarkers, stratifying patients within a particular disease group [70], or monitoring point mutations that frequently arise to confer drug resistance [71].

While direct analyte detection is now routine via MS, for complex samples, LC separation is required to gradually introduce the analytes into the mass spectrometer throughout the length of the gradient [58]. Using LC is time consuming and can be cumbersome when performing nano-flow applications. By contrast, Affi-BAMS greatly simplifies the sample complexity via a convenient, “disposable kit”-based approach that has been successfully deployed in the commercialization by many immunoassay technologies [11,72,73]. The kit-based approach for targeted proteomics applications has proven successful as it grants the ability to monitor a defined set of targets in large cohorts, whereas LC-MS is more suitable for discovery approaches to properly address the sample complexity of biological specimens. In our approach, we apply the advantage of the high-throughput scanning capability of MALDI MS to measure Affi-BAMS’ microarrays containing thousands of spots of bead-captured analytes. The microarrays are produced on standard-size microscope slides that fit the majority of current MALDI MS instruments and have flat surfaces, thereby eliminating the need for orthogonal laser beam geometry, which is a prerequisite for probing arrays of microwells [74].

Previously, reported platforms (e.g., SELDI and antibody microarrays) rely on highly reproducible chip surfaces, which often vary from batch to batch [75,76]. Affi-BAMS spatially separates each assay and allows independent optimization of affinity capture and detection of the target analytes. Avoiding the need for specialized chip surfaces eliminates a potentially significant source of analytical variability as long as the antibody-bead performance remains stable. By further improving conventional MALDI workflow with a standardized matrix application during the analyte elution conditions, we have eliminated a major source of analytical variation due to heterogeneous distribution of MALDI matrix within individual spots. Additional optimization has been achieved by reducing the diameter of spots to 500 µm (or 250 µm), which further increases the local concentration of target analyte. Higher target sensitivity and LOQ can be accomplished by consuming the entire spot to obtain a more accurate measurement of the analyte. In our hands, we found this method to be highly reproducible as depicted by the uniform microarray spots and the low variations in technical replicates of Affi-BAMS assays through our aforementioned studies.

Affi-BAMS has the potential to significantly lower the cost of proteomic assays, as expensive detection antibodies and fluorescent labels are eliminated from the workflow. Furthermore, several Affi-BAMS components including gaskets and slides may be reused to lower overhead cost. Additional improvements made during the method development (e.g., removal of the beads from the microarray before acquisition) further positions Affi-BAMS as a high throughput screening method, while also eliminating a potential source of instrument contamination in turn leading to reduction in the effort and cost of instrument maintenance.

Overall, the strength of Affi-BAMS derives from the ability to combine the specificity of an immunoassay with the precision and sensitivity of a mass spectrometer to provide a quantitative read out for select targets of interest. In our studies, we further demonstrate the ability to circumvent chromatographic separation and protein depletion methods in biofluids by concentrating target analytes on Affi-BAMS beads and microspots thereby increasing sensitivity for biomarkers and low abundant targets of interest [55]. With the unique high throughput workflow of Affi-BAMS, the same validated R&D assay that is used for investigational pre-clinical studies can be easily implemented as a diagnostic tool for screening of disease biomarkers as well as monitoring disease progression and therapeutic response. Moreover, as a targeted platform, deep insights into PTM modulation and crosstalk can be explored that will help expand insights into relevant biological regulation that may have been previously overlooked.

We demonstrate the use of Affi-BAMS for a variety of applications as a targeted analytical tool to understanding molecular mechanisms. These mechanisms, although very complex, can be dissected by honing in on specific protein targets of interest. Our studies show how multiple PTM sites can be resolved, quantified and localized on single proteins such as RPS6, ERK1/2 and 4EBP1. We also demonstrate how the assay can be easily adapted by modifying the sample preparation methods to shift the region of interest using alternative proteases to monitor flanking regions of the protein and gain deeper insights into adjacent PTMs or protein processing events. We applied this approach when examining histone marks and identified the large histone combinatorial crosstalk of acetylation, methylation and phosphorylation (among others) on histone H3 [77,78]. We were also able to identify the heterozygous point mutation that is well known on 4EBP1 in HCT116 (a colorectal cancer cell line) [64] and quantitated levels of total and specific phosphorylation site of 4EBP1 (pS101) [61] using the same antibody designed to recognize unmodified region of the C-terminus. Furthermore, we demonstrate the measurement of target analyte concentration via the use of SISCAPA reagents [41].

As new biomarkers are discovered, Affi-BAMS also provides an ability to re-analyze prior samples at a later time point (including patient samples). This decreases sample preparation and handling time and ensures consistency of input material. The Affi-BAMS method allows the flexibility to re-probe the same peptide samples using assays for new targets. This has been routinely performed in our validation experiments provided the flow through from the original sample is cryo-stored (or lyophilized, stored at −80 °C, and rehydrated upon use). In future work, development of the Affi-BAMS platform will focus on investigating individual peptide target stability and the ability to assay for specific protein targets after long-term storage, to establish ideal storage conditions. For new panels of target proteins, additional efforts will focus on configuring Affi-BAMS assays to investigate chromatin biology, including significant markers and enzymes which regulate them, to further increase the ability to crack the combinatorial histone code [79]. Assays will also be developed to identify novel peptide/protein biomarkers in liquid biopsy samples that have eluded many due to either low expression or complexity of PTM status. The novel Affi-BAMS platform, along with customizable assay panels, should aid the research community for straightforward MALDI MS quantification via targeted multiplexed microarrays.

## 4. Methods

### 4.1. Cell Culture

MKN-45 and HeLa cells were cultured in RPMI media with 10% fetal bovine serum (FBS), 1X Pen-Strep (Sigma, #P4333) to 75% confluence at 37 °C with 5% CO_2_. Prior to chemical treatment, cells were serum starved in RPMI media with 0.2% FBS and 1× Pen-Strep for 12 h. U2OS cells were cultured in DMEM media containing 10% FBS, 1× Pen-Strep to 75% confluency at 37 °C with 5% CO_2_. UT7epo-E cells were cultured in IMDM, 10% FBS, 1× Pen-Strep, and 2 U/mL EPO for 50+ passages.

### 4.2. Cell Culture Treatments

Su11274 (SU) and Staurosporine (ST) were used at a final concentration of 1 μM and 0.2 μM, respectively, in 0.05% DMSO for 2 h. H2O2 was used at a final concentration of 2 mM with a 30 min pre-treatment of 0.1 mM Na3VO4. Rapamycin was used at a final concentration of 1 mM for 2 h. SAHA treatment was used at a final concentration of 5 μM for a period of 24 h. The same volume of DMSO was used as controls for each treatment. UT7epo-E cells were collected (500× *g*, 8 min), washed twice in one-half culture volumes of 1% FBS, PBS (37 °C) and cultured for 20 h in 10% FBS, IMDM, 1× P-S (without EPO) and subsequently challenged with EPO (5 U/mL) for 15 min, or with PBS as a negative control.

### 4.3. MKN45 Rapamycin and SU11274/Staurosporine SILAC Labeling

Cells were grown for six passages in labeling media (Silantes #2800012000) containing either normal arginine and lysine or heavy arginine (+10, Silantes #201603902) and lysine (+8, Silantes #211603902) to 70% confluence. For the rapamycin inhibition study, light media cells were treated with DMSO (control) and heavy labeled cells were treated with rapamycin (inhibitor). For the kinase inhibitor study, light media cells were treated with SU11274 and Staurosporine (inhibitor) and heavy labeled cells were treated with DMSO (control). Equal quantities of total protein were combined at a ratio of 1:1 (Light: Heavy) for subsequent sample processing [45].

### 4.4. UT7epo-E Cells SILAC Labeling

UT7epo-E cells were plated at 1 × 10^5^ cells/mL (2 U/mL EPO) and expanded to generate cultures at ~7 × 10^5^ cells/mL. To pause EPO signaling, while heightening levels of unligated cell surface EPORs, EPO was then withdrawn as follows. Cells were collected (500× *g*, 8 min), washed twice in one-half culture volumes of 1% FBS, phosphate buffered saline (PBS, Thermo Fisher, #10010023) (37 °C) and cultured for 20 h at 7 × 10^5^ cells/mL in 10% FBS, IMDM, P-S (without EPO). Using 1 × 10^8^ cells per sample, UT7epo-E cells were then challenged with EPO (5 U/mL) for 15 min, or with PBS as a negative control. Cells were then immediately transferred to one-third volume of 2 °C PBS, incubated on ice for 5 min, and collected (2 °C, 500× *g*, 8 min).

### 4.5. Protein Concentration Determination

Protein concentration was performed using Bradford protein assay (Life Technologies, Carlsbad, CA, #23236) following the manufacturer’s protocol.

### 4.6. Preparation of Protein Lysates

Cells were washed twice with cold PBS. PBS was removed, and cells were scraped in cold Urea Lysis Buffer (9 M sequence grade Urea, 20 mM HEPES pH 8.0, 1 mM β-glycerophosphate, 1 mM sodium vanadate, 2.5 mM sodium pyrophosphate) 4 °C (careful not to let sit on ice since this will cause urea to precipitate out of solution). Cells were sonicated three times for 20 s each at 15 W output power with a 1 min cooling on ice between each burst. Sonicated lysates were centrifuged for 15 min at 4 °C at 20,000× *g*.

### 4.7. Peptide Sample Preparation

Protein digestions were carried out using standard methods for a variety of proteases (trypsin, chymotrypsin, Lys-C, Arg-C, Asp-N) as described by Giansanti et al. [60]. Protein lysates were collected and reduced with 4.5 mM DTT for 30 min at 40 °C. Reduced lysates were alkylated with 10 mM iodoacetamide for 15 min at room temperature in the dark. Samples were diluted 1:4 (*v:v*) with 0.2% ammonium bicarbonate (pH 8.0) and digested overnight with 10 µg/mL trypsin-TPCK (Promega) in 1 mM HCl. Digested peptide lysates were purified over SEP PAK Classic C18 columns (Waters, Richmond, VA, USA, #WAT051910) following manufacturer’s recommended protocol. Peptides were eluted with 60% acetonitrile in 0.1% TFA, and dried under vacuum in a lyophilizer (Virtis, SP Scientific), and stored at −80 °C for long-term storage.

### 4.8. Western Blotting

Protein Lysates were mixed with SDS-PAGE sample buffer (CST, #7723) and run on 4–20% gradient tris-glycine gels (Life Technologies). Proteins were transferred to nitrocellulose (Millipore, Billerica, MA) and blocked for 1h in 5% nonfat dry milk (Sigma) in TBST. Primary antibodies were incubated in 5% BSA in TBST overnight at 5 °C. Membranes were washed 4 times (15 min each) with TBST, incubated with anti-rabbit or anti-mouse secondary antibody (CST #5366 and #5470) for 1 h at room temperature in 5% milk TBST, washed 3 times (5 min each) with TBST, and developed on ChemiDoc MP imaging system (Bio-Rad, Hercules, CA, USA).

### 4.9. C-MET SIS Spike

Three replicates of 100 μg of MKN45 cell lysate was proteolytically digested with trypsin. Two of the three samples were spiked with either 1000 fmol or 100 fmol of C-MET SIS peptide (DMYDKEyySvHNk, y = phosphotyrosine, v = heavy valine (+6) and k = heavy lysine (+8)). A fourth mixture was prepared which contained only the SIS peptide at 10 fmol/μL.

### 4.10. SISCAPA Assay Sample Testing for BAMS Assay Workflow

SISCAPA sample preparation was carried out as described previously, with the following modifications [41]. Digested plasma samples were provided by SISCAPA Assay Technologies along with the PCI (Protein C Inhibitor) anti-peptide rabbit monoclonal antibody for Affi-BAMS bead preparation. The forward curve was generated by spiking a constant amount of SIS PCI peptide (1000 fmol/well) and varying a synthetic light peptide (10,000 fmol to 0.17 fmol) into 150 μL of trypsin digested human plasma to generate a 12-point curve with no spike of the light peptide in the 12th sample. The reverse curve was generated by spiking 150 μL of trypsin digested human plasma with a fixed amount of light peptide (1000 fmol/well) and varying the heavy SIS peptide tittered from 10,000 fmol to 0.06 fmol.

### 4.11. BAMS Assay—Bead Preparation

Each antibody was conjugated to NHS-activated magnetic agarose beads in a 10 μL slurry (7 μg of antibody to 100 agarose beads, 375–420 micron diameter) in PBS buffer at 4 °C overnight, followed by quenching with Tris HCl (100mM, pH 8.0) for 1h at RT. The following antibodies were used in this study: 4EBP1 (total, C-terminal, Cell Signaling Technology (CST) #9644), 4EBP1 (pT37 and pT46, CST #2855), 4EBP1 (pS65, CST #9451), 4EBP1 (pT70, CST #13396), C-MET (pY1234 and pY1235, CST #3077), RPS6 (pS235 and pS236, CST #4856), BAD (pS75, #5284), ERK1/2 (pT202 and pY204/pT185 and pY187), CST #4370), CTNNB1 (pS675, CST #4176), AKT1 (total, C-terminal, CST #9018), mTOR (pS2448, CST #5536), STAT3 (pY705, CST #9145) and H3 (acetyl-K9, Active Motif #91104). Unbound antibody was removed with three sequential 400 μL washes of cold PBS (2 min, 4 °C).

### 4.12. BAMS Assay—Target Peptide Binding

Individual target peptide enrichment was performed using 10–1000 µg of purified peptides with typically three replicate beads/targets. Multiplex target peptide enrichment was performed using 10–1000 µg of purified peptides with typically three replicate beads/target in a volume of 50–200 μL. Digested peptides were incubated in binding buffer (1M KCl, 100 mM Tris HCl in deionized water, pH 8.0) for a period of between 3–12 h at 4 °C. Beads were washed sequentially (2 min, 4 °C) in individual wells of a 48-well plate containing PBS (700 μL), Ammonium Bicarbonate Buffer (700 μL, 10 mM, pH 8.0) and deionized water (700 μL), respectively, to remove any nonspecifically bound peptides. Beads were by transferred between individual wells using a QuicPick magnetic bead handler (Bio-Nobile, #24001).

### 4.13. BAMS Assay—Target Peptide Elution

The washed Affi-BAMS beads are carefully transferred to the hydrated wells of appropriate sample chamber using a magnetic bead handler. The Affi-BAMS beads settle into the micro-wells with gentle agitation followed by centrifugation in a swinging rotor bench top centrifuge (5 min, 200× *g*). After centrifugation, the sample chamber gasket is removed, leaving the micro-well gasket fixed in place on the slide. Residual water is removed by padding on a dry towel with a magnet placed on the opposite side to hold the Affi-BAMS beads in place within the micro-wells. The bead array is exposed to an aerosol of elution buffer using a Matrix Sprayer, containing 0.5 mg/mL α-cyano-4-hydroxycinnamic acid (CHCA) in 50% acetonitrile and 0.4% trifluoroacetic acid (TFA). Robotic liquid sprayer (iMatrixSpray) for depositing MALDI matrix solutions was obtained from Tardo GmbH (Subingen, Switzerland). The aerosol is applied to the surface of the plate with the micro-well gasket and Affi-BAMS beads facing the MALDI matrix sprayer. Application of the aerosolized elution buffer allows reproducible delivery of small, precisely measured amounts of matrix-containing solution uniformly distributed into every micro-well across the entire surface of the slide without overfilling the wells. The application of the elution buffer is optimized such that the rate of the solution deposition onto the slide is closely matched to the rate of the solvent evaporation from the slide. The spraying protocol was designed and adjusted based on the rate of matrix solution deposition according to the ambient humidity level as measured by a hygrometer, such that the rate of the matrix solution deposition into the microwells is approximately equivalent to the rate at which the solvent of the matrix solution is escaping from the microwell via evaporation. For ambient humidity levels between 35% and 65% of relative humidity and indoor temperature between 20 and 28 °C, the matrix deposition parameters of iMatrixSpray were set as follows: Height: 60 mm; Line Distance: 0.5 mm; Speed: 60 mm/s; Density: 5 μL/cm2; Number of cycles: 10; Delay: 0 sec; Spray area width: 80 mm; Spray area depth: 30 mm. For ambient humidity levels between 15% and 35%, the speed parameter was changed to 90 mm/s, the other parameters remaining the same. For ambient humidity levels between 65% and 85%, the speed parameter was changed to 40 mm/s and the line distance parameter to 0.3 mm, the other parameters remaining the same. In all cases, the MALDI matrix solution contained 5 mg/mL of α-hydroxy cinnamic acid (CHCA), 0.4% (*v/v*) of TFA and 50% (*v/v*) of acetonitrile.

To prevent splattering of the matrix solution from the microwells, the pressure setting for the carrier nitrogen gas of iMatrixSpray was set to 0.09 MPa, slightly below the lowest recommended setting of 0.1 MPa. The MALDI matrix sprayer delivers elution buffer to the surface of the plate for approximately 15 min to ensure efficient release of the captured peptides. The low pH of elution buffer causes captured peptides to dissociate from each Affi-BAMS bead while the sidewalls of the micro-well gasket prevent the eluted peptides from diffusing into adjacent wells. Once the elution cycle is complete, residual solvent evaporates and the released peptides co-crystallize and become incorporated into the MALDI matrix in confined spots located at the bottom of micro-wells and the surface of the slide. Once the matrix is dry, the silicone gasket is lifted off the slide and any remaining dry agarose beads are removed by compressed air leaving an array of spots containing purified and concentrated target peptides for subsequent MALDI MS measurement.

### 4.14. MALDI TOF Linear Instrument Settings

Matrix Assisted Laser Desorption Ionization Time of Flight (MALDI TOF) MS data was acquired on Bruker Daltonics (Billerica MA) Autoflex Speed MALDI TOF-TOF mass spectrometer using FlexControl v.3.4 software and low mass range data acquisition methods supplied by the manufacturer. Unless otherwise indicated, mass spectra were acquired in the positive linear mode, 750–7000 m/z mass range using the laser repetition rate of 2 kHz. Between 2000 and 10,000 single shot spectra were collected from individual microarray spots using the random walk method. The instrument laser power was typically between 20–50%, laser attenuator offset 30%, attenuator range 20%. The voltage settings were 19.50 kV (ion source 1), 18.35 kV (ion source 2) and 6.0 kV (lens). The pulsed ion extraction was 130 ns. The detector gain voltage was 4.0X or 2910 V.

### 4.15. MALDI TOF Reflector Instrument Settings

In the positive reflector mode, the spectra were measured in the 750–5000 m/z mass range using the laser repetition rate of 2 kHz. The voltage settings were 19.00 kV (ion source 1), 16.55 kV (ion source 2), 8.45 kV (lens), 21.00 kV (reflector), 9.65 kV (reflector 2). The pulsed ion extraction was 100 ns. The detector gain voltage was 8.1X or 1919 V. The digitizer sampling rate was 4.00 GS/s.

### 4.16. MALDI TOF LIFT Instrument Settings

In the MS-MS mode, the spectra were collected using the LIFT method provided by the manufacturer at the laser repetition rate of 2 kHz (parent ions) and 200 Hz (fragment ions). The voltage settings were 6.00 kV (ion source 1), 5.30 kV (ion source 2), 3.00 kV (lens), 27.20 kV (reflector), 11.70 kV (reflector 2), 19.00 kV (lift 1), 4.20 kV (lift 2). The pulsed ion extraction was 100 ns. The reflector gain voltage was 19.4X or 2051 V. The digitizer sampling rate was 2.00 GS/s.

### 4.17. MALDI TOF Calibration

The instrument was externally calibrated in cubic enhanced mode using a mixture of peptides derived from trypsin-digested CAM-modified bovine serum albumin (BSA) supplemented with human insulin (Sigma, I6383). Molecular weights of the calibration peptides spanned a range between 800 and 8000 Da.

### 4.18. MALDI TOF Acquisition

Prior to MS data acquisition, analyte-containing spots within each microarray were identified by visual inspection and their x-y coordinates submitted to AutoXecute module of FlexControl. The analyte-containing spots were identified based on their characteristic appearance (co-crystalized, crescent shaped MALDI matrix and analyte) due to the presence of inner areas devoid of the MALDI matrix that coincide with the locations of Affi-BAMS beads in the corresponding microwells. MS data was subsequently acquired from the selected spots in AutoXecute mode. Mass spectra collected from each spot were averaged from each spot and saved as individual spectra with the corresponding x-y coordinate location appended to the file name.

### 4.19. MALDI TOF Analysis

Mass spectra were processed and analyzed using FlexAnalysis v.3.4 software. Peaks were detected that had a signal-to-noise ratio of at least 3. In some cases, baseline subtraction procedure was applied to individual mass spectra. Unless otherwise indicated, peaks in the mass spectra are labeled using either average or monoisotopic m/z values using the peak picking algorithms available in the FlexControl software. Mmass was used for data review [80].

### 4.20. MALDI TOF MS/MS Peptide Identification

Peptide sequencing data was produced using the LIFT method and analyzed using the online program MS-Tag (ProteinProspector, University of California San Francisco). The MS-Tag settings were as follows: database: SwissProt 2017.11.01; taxonomy: Homo Sapiens; digest: no enzyme; constant mods: carbamidomethyl (C); variable mods: acetyl (K), deamidated (R), methyl (K), dimethyl (K), trimethyl (uncleaved K), phospho (ST), Gln->pyro-Glu (N-terminal Q), oxidation (M); parent ion tolerance: 200 ppm; fragment tolerance: 0.8 Da; max mods: 4; instrument: MALDI-TOFTOF.

## Figures and Tables

**Table 1:** Rapamycin treatment in MKN45 Cells. MKN45 cells were SILAC labeled (light = DMSO, heavy = rapamycin; K + 8 and R + 10) and treated with either DMSO or 1mM rapamycin for 2 h. A multiplexed Affi-BAMS assay was conducted for total and phospho-specific targets. The calculated mass of the light and observed light and heavy SILAC pairs are annotated in the table along with the heavy incorporated residues (blue) and phosphorylated residues (red). The average ratio (H:L) and coefficient of variation (C.V.) is provided for each Affi-BAMS assay.

Accession	Protein	Site/Total	Average Ratio (H:L)	C.C. (%)	Peptide	Calc. Light m/z	Light m/z	Heavy m/z
Q13541	4EBP1	Total	1.22	2.72	K.RAGGEESQFEMDI.-	1468.637	1468.83	1478.86
P42345	mTOR	p(S2448)	0.15	5.98	R.TRTDsYSAGQSVEILDGVELGEPAHK.T	2839.315	2839.25	2856.99
p35222	CTNNB1	p(S675)	1.73	3.53	K.RLsVELTSSLFR.T	1487.762	1487.21	1507.40
P31749	AKT1	Total	2.89	11.07	R.RPHFPQFSYSASGTA.-	1652.782	1652.58	1662.34
Q13541	4EBP1	p(T37 or T46)	3.06	9.09	R.VVLGDGVQLPPGDYSTtPGGTLFSTTtPGGTR.I	3283.599	3282.50	3302.57
Q13541	4EBP1	p(T37 or T46)	0.25	8.57	R.RVVLGDGVQLPPGDYSTtPGGTLFSTtPGGTR.I	3363.566	3363.18	3383.25
Q13541	4EBP1	p(T65 or T70)	0.01	7.61	R.NsPVTKtPPRDLPTIPGVTSPSSDEPPMEASQSHLR.N	3985.840	3985.74	4003.29
Q92934	BAD	p(S75)	0.33	10.67	R.HSsYPAGTEDDEGMGEEPSPFR.G	2474.944	2474.96	2484.94
P62753	RPS6	p(S235 & S236)	0.04	6.96	R.RLssLRASTSKSESSQK.-	2091.887	2091.57	2128.3

**Table 2:** EPO Challenge in UT7epo-E cells. SILAC labeled UT7epo-E cells (light= −EPO, heavy= +EPO; K + 8 and R + 10) were challenged with +/− 5 U/mL EPO for 15 min. The calculated mass of the light and observed light and heavy SILAC pairs are annotated in the table along with the heavy incorporated residues (bold blue) and phosphorylated residues (lower case red). The average ratio (H:L) and coefficient of variation (C.V.) is provided for each Affi-BAMS assay.

Accession	Protein	Site/Total	Average Ratio (H:L)	C.C. (%)	Peptide	Calc. Light m/z	Light m/z	Heavy m/z
P42345	mTOR	p(S2448)	9.29	17.13	R.TRTDsYSAGQSVEILDGVELGEPAHK.T	2893.315	2839.46	2857.96
Q92934	BAD (0XC)	p(S75)	4.11	9.70	R.HSsYPAGTEDDEGMGEEPSPFR.G	2474.944	2474.93	2484.93
Q92934	BAD (0XC)	p(S75)	4.31	1.84	R.SRHSsYPAGTEDDEGMGEEPSPFR.G	2718.077	2717.97	2738.05
P62753	RPS6 (2p, 2XC)	p(S235 & S236)	145.75	23.87	R.RLssLRASTSK.S	1365.629	1365.68	1393.72
P62753	RPS6 (2p, 3XC)	p(S235 & S236)	21.67	28.43	R.RLssLRASTSKSESSQK.-	2011.921	2011.94	2048.00
P62753	RPS6 (3p, 3XC)	p(S235 & S236)	229.47	40.42	R.RLssLRASTSKSESSQK.-	2091.887	2091.61	2127.43
P62753	RPS6 (2p, 4XC)	p(S235 & S236)	12.54	34.98	R.RRLssLRASTSKSESSQK.-	2168.022	2168.27	2214.05
P62753	RPS6 (4p, 3XC)	p(S235 & S236)	111.60	21.81	R.RLssLRASTSKSESSQK.-	2171.854	2171.53	2207.25
P62753	RPS6 (3p, 4XC)	p(S235 & S236)	413.93	53.49	R.RRLssLRASTSKSESSQK.-	2247.988	2247.98	2293.76
P62753	RPS6 (5p, 3XC)	p(S235 & S236)	76.67	43.30	R.RLssLRASTSKSESSQK.-	2251.820	2251.97	2287.42
P62753	RPS6 (4p, 4XC)	p(S235 & S236)	257.49	63.18	R.RRLssLRASTSKSESSQK.-	2327.955	2327.53	2373.84
P62753	RPS6 (5p, 4XC)	p(S235 & S236)	214.19	53.55	R.RRLssLRASTSKSESSQK.-	2407.921	2408.01	2453.53
P40763	STAT3	p(Y705)	19.54	11.65	K.YcRPESQEHPEADPGSAAPyLK.T	2582.102	2582.14	2600.31
P28482	ERK2(1p)	p(T185/Y187)	38.80	9.89	R.VADPDHDHTGFLtEYVATR.W	2223.970	2223.79	2234.35
P28482	ERK2(2p)	p(T185/Y187)	4.19	20.73	R.VADPDHDHTGFLtEyVATR.W	2303.937	2303.82	2314.38
P27361	ERK1(1p)	p(T202/Y204)	90.97	28.05	R.IADPDHDHTGFLtEYVATR.W	2252.002	2252.51	2262.34
P27361	ERK1(2p)	p(T202/Y204)	4.97	8.56	R.IADPDHDHTGFLtEyVATR.W	2331.968	2331.50	2341.52

## Supplementary Materials

Supplementary materials can be found at https://www.mdpi.com/1422-0067/21/6/2016/s1.
