# Supplementary material for: Affinity-Bead Assisted Mass Spectrometry (Affi-BAMS): A Multiplexed Microarray Platform for Targeted Proteomics
Source: Int J Mol Sci. 2020 Mar 16;21(6):2016. doi: 10.3390/ijms21062016 (PMC7139916; doi:10.3390/ijms21062016)
Supplement: Supplementary file 1 [file ijms-21-02016-s001.zip › ijms-728665-for publish supplementary/Supplemental Material.docx]

**
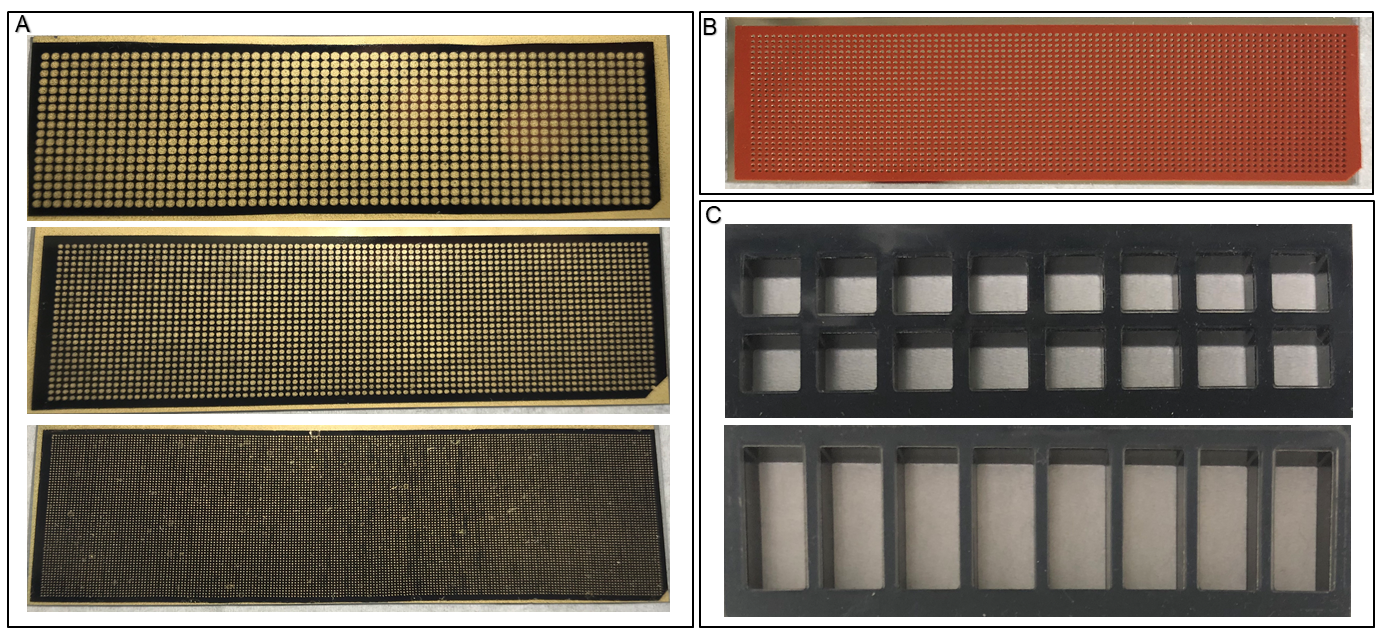
Supplemental Figure 1. Affi-BAMS Components as Engineered Microslide Arrays, and Corresponding Gaskets and Sample Chambers:** Affi-BAMS has several components in order to achieve spatially confined elution of bead-bound targets for MALDI MS analysis. An array of spatially separated spots containing eluted targets co-crystalized with the MALDI matrix is produced according to the packing density and the well diameter elastomer gasket used (Panel A). The elastomer gasket attached to a microscope slide is utilized to spatially separate and confine each Affi-BAMS bead for target elution (Panel B), while a multi-chamber frame allows for beads from individual samples to be separated (Panel C).


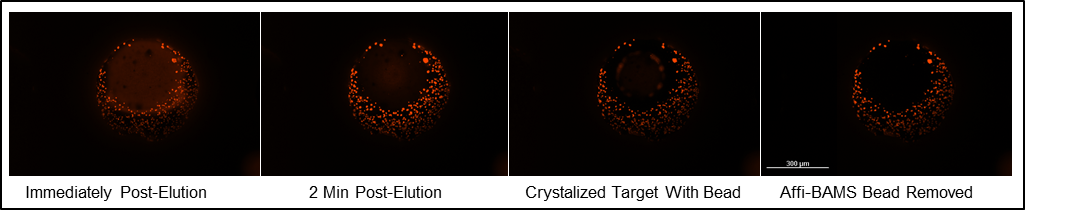
**Supplemental Figure 2. Affi-BAMS Target Peptide Elution:** Synthetic peptide Texas Red-RAGGEESQFEMDI-OH containing an amino acid sequence corresponding to the C-terminus of 4EBP1 was from New England Peptides (Gardner MA). The peptide was dissolved in PBS to the concentration of 5µM. The peptide-containing solution was incubated with Affi-BAMS beads conjugated to an antibody specific for 4EBP1. The beads were subsequently rinsed and placed on a microwell array plate consisting of a previously described elastomer gasket containing an array of 500-micron diameter through-holes that was attached to an ITO-coated glass microscope slide (Bruker Daltonics, Billerica MA). The MALDI matrix application was performed as described in the Methods section. The bead array was removed from the matrix sprayer immediately after the matrix application cycle was complete and before complete solvent evaporation. A series of images of a single well were acquired using fluorescent digital microscope (Cytation 3, Biotek, Winooski VT) in the Texas Red channel (586 nm). Images were collected at sequential intervals as noted. The final image was recorded after the gasket was separated from the ITO slide and dry Affi-BAMS bead removed from the slide surface. Note that the gasket and bead removal procedures did not displace any matrix crystals from the spot.


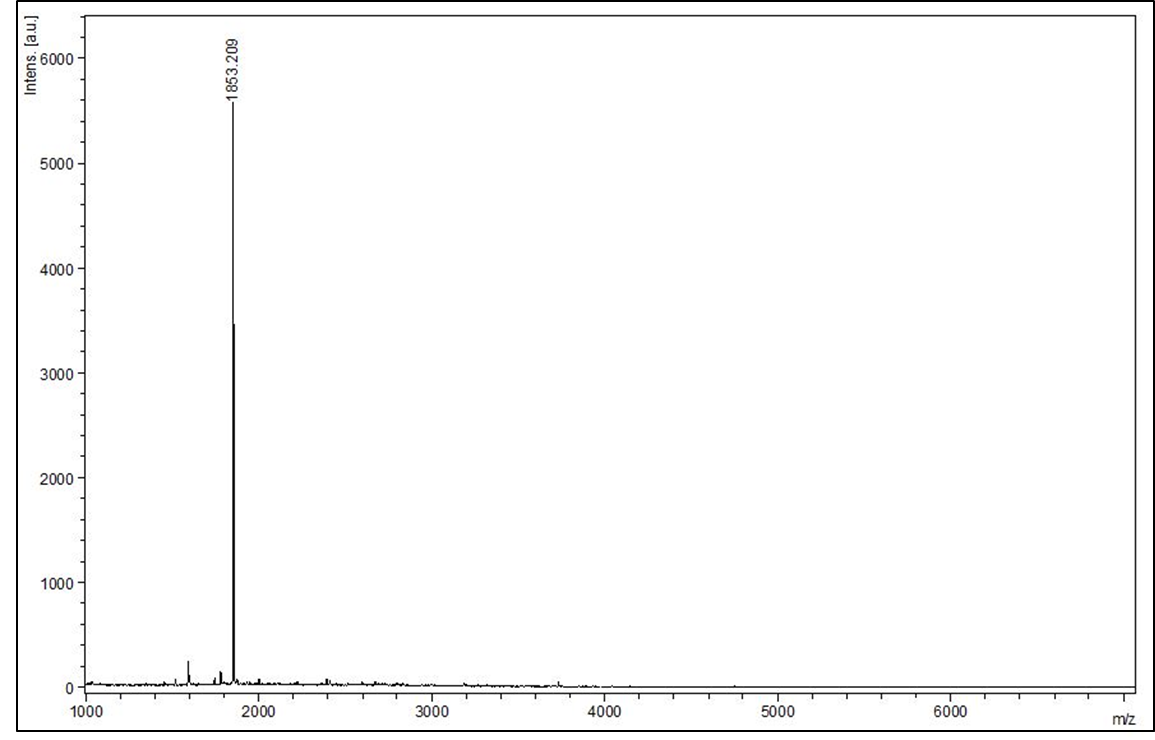
**Supplemental Figure 3. Detection and Quantification of PTMs on C-MET:** Control sample was prepared using C18 purified peptides from a tryptic digest of MKN45 cells. An Affi-BAMS assay for C-MET (pY1234 & pY1235) was performed, and we acquired a MALDI MS spectrum for the captured and eluted target peptides. An *in silico* tryptic digest of C-MET produces the following peptides from zero to two missed cleavages: 1) K.E**YY**SVHNK.T, 2) K.E**YY**SVHNKTGAK.L and 3) R.DMYDKE**YY**SVHNK.T with pY1234 & pY1235 highlighted in BOLD and underlined. The calculated masses (MH+, average) for each of the peptides listed above are 1200.087, 1557.499 and 1852.815 m/z, respectively. The data collected from the MALDI MS spectrum shows a single dominant peak at 1853.21 m/z corresponding to the two missed cleavage products listed above that is presumably due to the presence of the KE residues and also the dually phosphorylated pY1234 and pY1235 residues forcing missed cleavages.


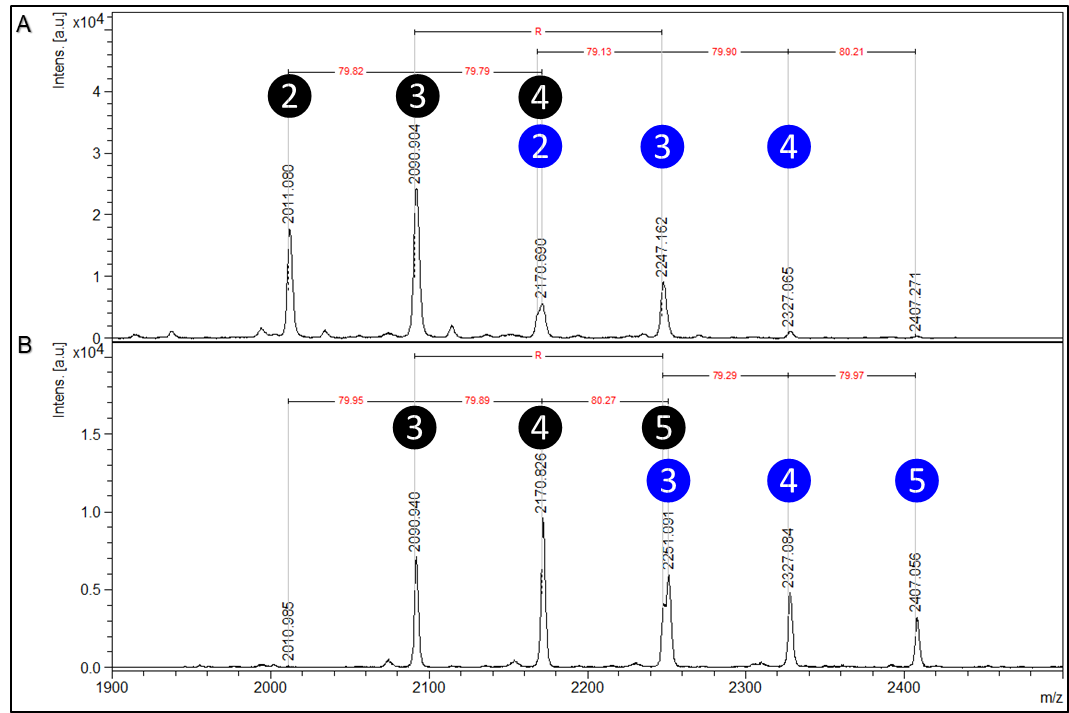


**Supplemental Figure 4. Detection and Quantification of Multiple PTMs on RPS6:** Samples were prepared using C18 purified peptides from a tryptic digest of MKN45 cells that was treated with either DMSO (Panel A) or 2mM H_2_O_2_ for 20 minutes (Panel B). Comparing the two MALDI MS spectra from each condition, we observe a series of common peaks between 2000 and 2500 m/z, with some distinct differences among the relative peak intensities. The first series of masses are associated with the base sequence that spans amino acids 223-249, R.RL**SS**LRASTSKSESSQK.-, containing two (2011.915 MH+), three (2091.776 MH+) and four (2171.798 MH+) phosphorylation’s (with the pS235 & pS236 highlighted in bold and underlined). The second series of masses are associated with the base sequence that spans amino acids 222-249, R.RRL**SS**LRASTSKSESSQK.-, containing two (2168.337 MH+), three (2248.100 MH+), four (2328.034 MH+) and five (2407.774 MH+) phosphorylation’s (with the pS235 & pS236 highlighted in bold and underlined). Looking at the 20-minute peroxide treatment (B), there is an increase of phosphorylation status. The MALDI MS spectrum for the RL**SS**LRASTSKSESSQK peptide shows between 2 to 5 phosphorylation’s, with the relative intensity of the dually phosphorylated species substantially lower than what is observed in the DMSO. Similarly, the MALDI MS spectrum for the RRL**SS**LRASTSKSESSQK peptide also shows higher order phosphorylation (between 3 to 5) as observed from the relative intensities of the corresponding masses and the reduction of the dually phosphorylated form of the peptide.


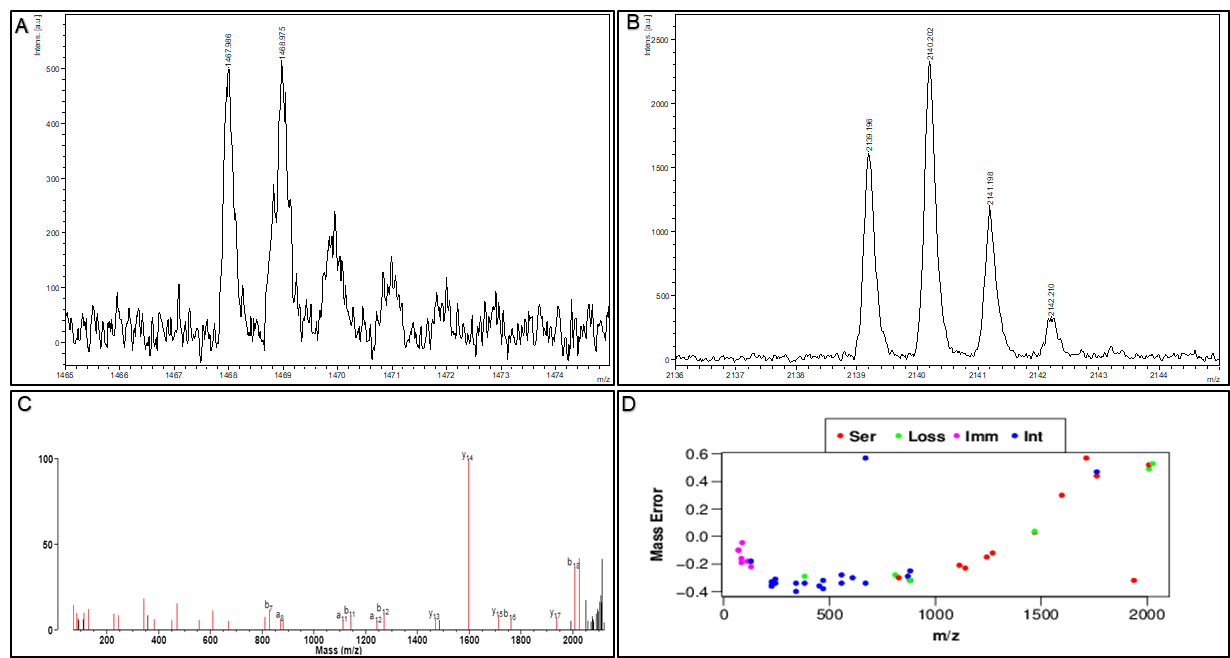
**Supplemental Figure 5. Detection of 4EBP1 in Human Serum:** Human serum was purchased from Innovative Research, Inc (Novi, MI) and pooled into one sample. 100µL of human serum (~7,000 µg of total soluble protein) was digested with trypsin, and Affi-BAMS assay for 4EBP1(total, C-terminus) was performed (see Methods). An *in silico* tryptic digest of 4EBP1 produces the following peptides from zero to two missed cleavages: 1) R.AGGEESQFEMDI.- , 2) K.RAGGEESQFEMDI.- and 3) R.NSPEDKRAGGEESQFEMDI.- with calculated masses (MH+, average) of 1313.391, 1469.579 & 2140.260 m/z, respectively. The 4EBP1 peptide RAGGEESQFEMDI (Panel A) and NSPEDKRAGGEESQFEMDI (Panel B) is observed within the serum sample. An MS/MS spectrum was collected for the most intense peak in panel B, 2140.20 (m/z, z=1), and was searched through Protein Prospector and identified as the NSPEDKRAGGEESQFEMDI peptide to 4EBP1 (Panel C and D).

**
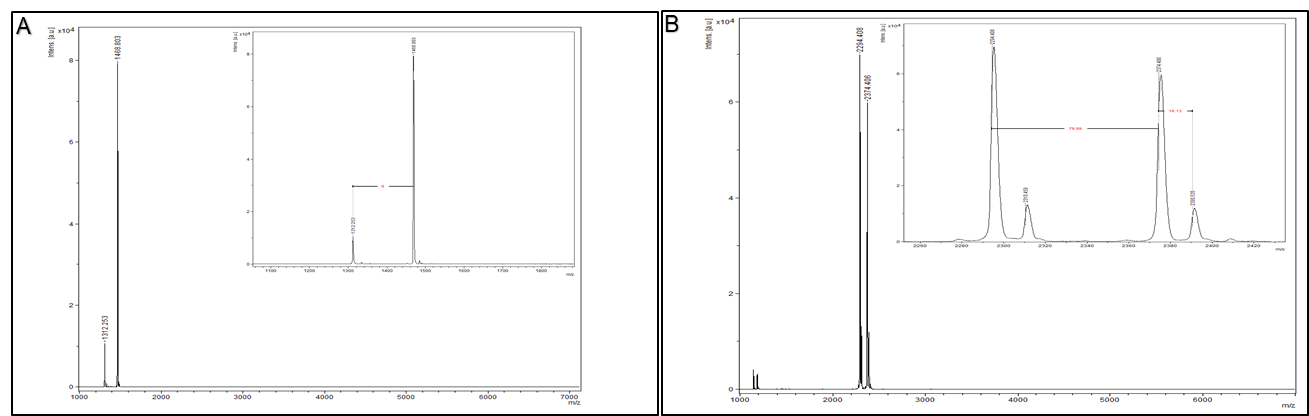
Supplemental Figure 6. Utilizing Differential Proteases as an Orthogonal Approach:** Control sample was prepared using C18 purified peptides from either a tryptic or chymotryptic digest of MKN45 cells (untreated). A full scan MALDI MS spectrum (750 – 7000 m/z) was collected in linear mode from the Affi-BAMS assay for 4EBP1 (tryptic conditions), showing 1312.25 & 1468.80 (m/z, z=1) as the two most predominant peaks (Panel A) as expected for the tryptic peptide for the zero and one missed cleavage products. Under chymotryptic conditions, we observe the following two peptides from the 4EBP1 (total, C-terminus) Affi-BAMS assay: L.RNSPEDKRAGGEESQFEMDI.- and L.RN**S**PEDKRAGGEESQFEMDI.-, with the calculated masses (MH+, monoisotopic) of 2295.031 and 2374.997, respectively. A full scan MALDI MS spectrum (750 – 7000 m/z) was collected in linear mode from the Affi-BAMS assay for 4EBP1 (chymotryptic conditions), showing 2294.41 & 2374.41 (m/z, z=1) as the two most dominant peaks (Panel B), corresponding to the chymotryptic peptide with and without phosphorylation at Serine-101.


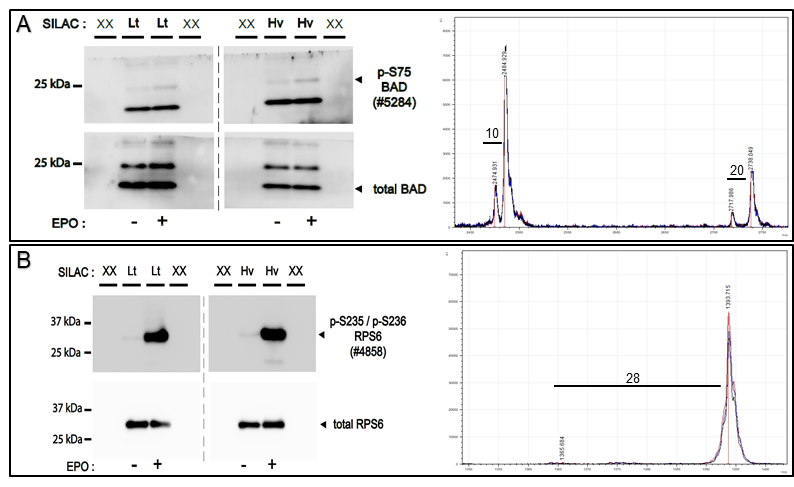


**Supplemental Figure 7. EPO Challenge in UT7epo-E cells:** SILAC labeled UT7epo-E cells (forward labeled [light= -EPO, heavy= +EPO] and reverse labeled [light= +EPO, heavy= -EPO]; K+8 & R+10) were challenged with +/- 5U/mL EPO for 15 minutes. Western blots were conducted to validate target’s response for both light and heavy SILAC labeled cells as well as reverse labeled cells to confirm Affi-BAMS assays’ results (lt=Light, Hv=Heavy, XX designates empty lane. The MALDI MS for select targets in the multiplexed Affi-BAMS assay are shown for the forward labeled pair. Mass shift due to the heavy labeling of residues is notated as well as phosphorylation shifts. Raw files were imported into mMass and normalized between replicates on the most intense peak to generate ratios between the light and heavy SILAC pairs.


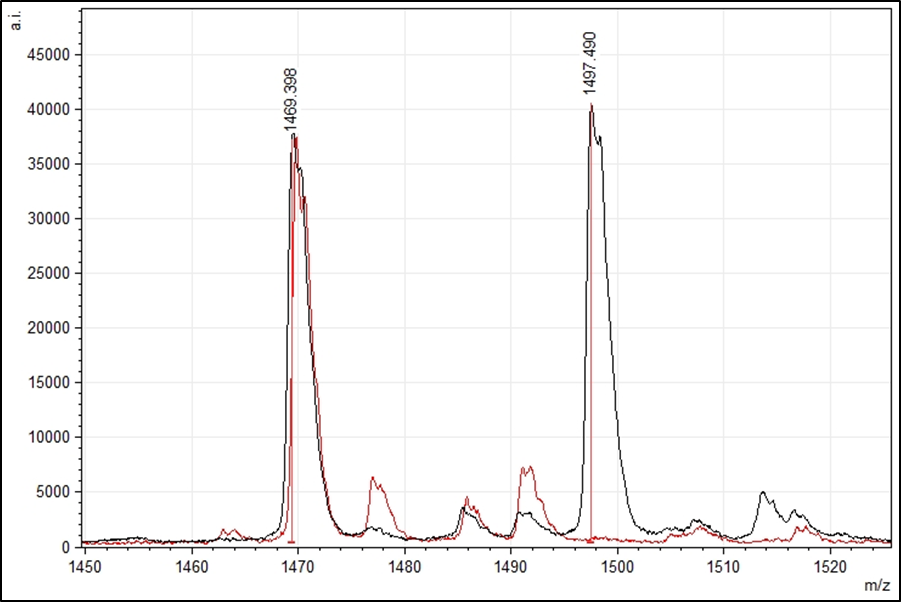


**Supplemental Figure 8. Affi-BAMS assay for 4EBP1 in MKN45 and HCT116 cells:** Affi-BAMS assays for 4EBP1 (C-terminal, Total) in MKN45 (red spectrum) and HCT116 cells (black spectrum) denote the point mutation that is found only in HCT116 cells. The HCT116 spectrum highlights the R**A**GGEESQFEMDI (1468.637 m/z) and R**V**GGEESQFEMDI (1496.669 m/z) peptide (A106V mutation) in a heterozygous form as a delta change of 28 m/z. The MKN45 spectrum only identifies the R**A**GGEESQFEMDI (1468.637 m/z) peptide, as expected.
